# Integrated Computational Study Prioritizes Drug-like Para-Flavonoids as Candidate SARS-CoV-2 M^pro^ Inhibitors

**DOI:** 10.3390/cimb48090891

**Published:** 2026-08-31

**Authors:** Jawaher H. Alqahtani, Mohammed Ouachekradi, Bahia Abdelfattah, Amal Elrherabi, Moneerah J. Alqahtani, Joe Miantezila Basilua, Oussama Khibech, Mohamed Bouhrim

**Affiliations:** 1 Department of Pharmacognosy, College of Pharmacy, King Saud University, P.O. Box 2457, Riyadh 11451, Saudi Arabia; 2 Laboratory of Applied Chemistry and Environment, Faculty of Science, Mohammed First University, BP 717, Oujda 60000, Morocco; 3 Laboratory of Physical Chemistry of Materials, Natural Substances and Environment, Chemistry Department, Faculty of Sciences and Techniques of Tangier, Tangier 90090, Morocco; 4 Laboratory for Research in Life and Drug Sciences (LRSVM), Pharmacology, Toxicology, and Applied Plant Sciences, Faculty of Medicine and Pharmacy, Mohammed First University, BP 717, Oujda 60000, Morocco; 5 BioSTM—UR 7537 Biostatistics, Processing and Modelling of Biological Data, Paris Cité University, 75006 Paris, France; 6 Biological Engineering Laboratory, Faculty of Sciences and Techniques, Sultan Moulay Slimane University, P.O. Box 523, Mghila, Beni Mellal 23000, Morocco

**Keywords:** SARS-CoV-2, antivirals, flavonoid, ADME, GROMACS, density-functional calculations, SDG3

## Abstract

Emerging SARS-CoV-2 variants highlight the need for orally active, low-toxicity antivirals. We designed seven para-substituted flavonoid hybrids (M1–M7) against the main protease (M^pro^). In silico ADME filtering revealed zero Lipinski, Veber, or Ghose violations, a SwissADME bioavailability score of 0.55, and selected favorable predicted absorption and transporter endpoints relative to lopinavir, without implying measured pharmacokinetic superiority. ProTox-III indicated that amino and nitro substitution increased predicted genotoxicity liabilities, whereas cyano and methoxy substitution reduced selected endocrine-related signals. AutoDock Vina docking to M^pro^ (PDB 9C8Q; redocking RMSD 0.316 Å) ranked the nitro analogue M6 first among the designed compounds (−8.1 kcal mol^−1^), with contacts involving His41 and neighboring active-site residues. During the 100 ns GROMACS simulations, the protein backbone remained stable, whereas M6 adopted a late reoriented pose that was retained in the active-site region and supported by late-window per-residue energetic contributions. DFT calculations at the B3LYP/6-311G(d,p) level identified the narrowest HOMO-LUMO gap (3.44 eV) and highest electrophilicity (ω = 6.2 eV) for M6. Overall, M6 is prioritized as a computational lead requiring M^pro^ inhibition, antiviral, and cytotoxicity validation.

## 1. Introduction

**Scheme 1:**
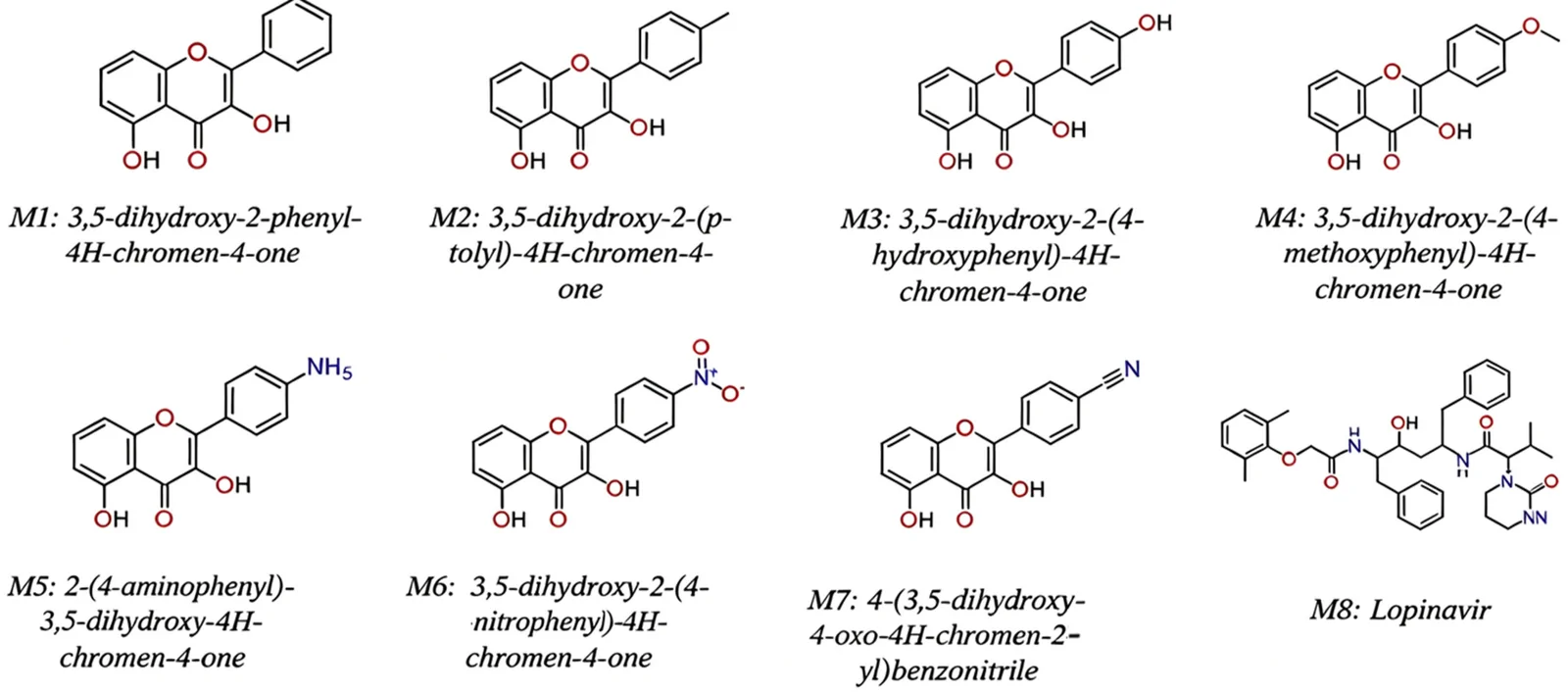
Chemical structures of the seven para-substituted flavonoid hybrids (M1–M7) and the reference inhibitor lopinavir (M8).

The unprecedented global spread of SARS-CoV-2 has galvanized medicinal-chemistry efforts to discover small-molecule antivirals that retain potency as the virus continues to mutate [1,2,3,4,5]. Although vaccination campaigns curb transmission, breakthrough infections and immune evasion by emerging variants underscore the need for orally available drugs that act independently of neutralizing antibodies [6,7]. Viral proteases, and in particular the cysteine main protease (M^pro^, nsp5), have become prime therapeutic targets because they orchestrate the proteolytic release of non-structural proteins required for replication yet share no close human homologues, minimizing off-target liabilities [8,9]. First-generation M^pro^ inhibitors such as nirmatrelvir validate this strategy, but their covalent peptidomimetic cores suffer from metabolic lability, high molecular weights, and a dependence on pharmacokinetic boosters that complicate long-term therapy [10,11]. Consequently, attention has turned to more compact, non-peptidic scaffolds capable of achieving high affinity without sacrificing drug-like properties [12]. Flavonoids are particularly attractive: their rigid, π-rich frameworks engage active-site residues through hydrogen bonding, π-π stacking, and cation-π interactions, while their biosynthetic accessibility facilitates rapid derivatization [13,14,15]. Yet unmodified flavones and flavonols often display poor aqueous solubility and rapid phase-II conjugation, limiting systemic exposure [16]. Recent computational screens have shown that strategic substitution on the B-ring can modulate polarity, electronic density, and interaction geometry, thereby improving both enzymatic binding and predicted pharmacokinetics [17,18]. Electron-withdrawing groups such as nitro or cyano have been reported to strengthen contacts with the catalytic dyad His41/Cys145, whereas electron-donating methoxy or amino groups may attenuate efflux susceptibility and alter cytochrome-P450 liability [19,20,21]. Parallel advances in in silico methodologies now permit rapid triage of large libraries [22]: SwissADME and ADMETlab forecast absorption, efflux, and metabolic stability; ProTox-III anticipates organ-specific toxicities from SMILES strings; high-resolution docking protocols validated by redocking RMSD values below 2 Å provide reliable binding poses; and explicit-solvent molecular-dynamics simulations reveal the temporal resilience of ligand–protease complexes. Coupled with frontier-orbital and reduced-density-gradient analyses from density-functional theory, these tools deliver a multiscale view that links electronic structure to macroscopic drug behavior. In this study, we exploit that integrative computational pipeline to scrutinize a focused panel of para-substituted flavonoid hybrids (Scheme 1), profiling their pharmacokinetic readiness, safety margins, binding energetics, and dynamic stability in the M^pro^ pocket, to identify chemically tractable leads for next-generation anti-COVID therapeutics. Within this design framework, para-substitution was used as a controlled perturbation of B-ring electronics and hydrogen-bonding capacity. Prior medicinal-chemistry work shows that B-ring architecture can markedly alter flavonoid target recognition [4,23,24],whereas extract-level profiling illustrates the compositional complexity that can obscure substituent-specific effects in heterogeneous flavonoid matrices [25]. The matched M1–M7 series therefore isolates para-substituent effects more directly than complex extracts. Computational drug-repurposing frameworks broaden candidate discovery but remain complementary to target-specific structural and experimental validation.

## 2. Materials and Methods

### 2.1. Pharmacokinetic Analysis Using Computational Tools

A compound’s pharmacokinetic profile absorption, distribution, metabolism and excretion (ADME) is dictated by a cascade of biochemical and physiological events that determine its fate in vivo. Precise knowledge of these parameters clarifies the mechanisms governing absorption, systemic distribution, metabolic transformation and ultimate elimination [26]. In silico strategies have become indispensable for analyzing and forecasting these properties: they simulate membrane permeability and biomolecular interactions during the absorption-to-excretion continuum while probing structural stability throughout metabolism [27]. In the present study, candidate molecules were first sketched in ChemDraw 25.0 and exported as SMILES strings. These SMILES representations were then submitted to SwissADME [28] and ADMETlab3 [29] for comprehensive pharmacokinetic assessment. ADMETlab classification endpoints were interpreted as model probabilities, whereas regression endpoints were interpreted in their reported transformed units.

### 2.2. Prediction of Toxicity Using ProTox-III

Prospective toxicity profiling was performed with the ProTox-III web server following its recommended workflow [30]. The submitted SMILES strings were converted into probability-based predictions for organ toxicity, toxicological endpoints, and molecular initiating events. In the radar plots, blue points represent the predicted probability for the query compound, while orange points represent the mean probability of active compounds in the corresponding training set. Predictions below the platform reporting threshold (generally 0.70) may be omitted; therefore, the displayed percentages are confidence estimates rather than clinical incidence rates or universal toxicity cutoffs. The accompanying classifications provide a multidimensional screening profile but require experimental confirmation [31].

### 2.3. AutoDock Vina: Preparation, Configuration, and Validation of the Docking Protocol

AutoDock Vina, integrated with the AutoDock Tools (ADT) interface v 1.5.7 [32], was used to perform all docking simulations. Before docking, the protein–ligand complex was prepared in line with the AutoDock suite guidelines: crystallographic water molecules were removed, requisite polar hydrogens were added, and appropriate atomic charges were assigned. Discovery Studio 2025 was likewise employed for protein preprocessing (eliminating redundant waters, applying structural corrections, etc.) and, post-docking, for extracting poses and analyzing ligand–receptor interactions [33]. Each ligand was initially sketched and geometry-optimized in ChemDraw, then subjected to energy minimisation [34]. The resulting structures were converted to PDBQT format in ADT 1.5.7 to ensure geometries and charge states compatible with Vina. The search space was defined by a 34 × 26 × 30 Å^3^ grid box centered on the active site (center_x = −14.687, center_y = 15.103, center_z = 0.945). Docking parameters were set to ten output modes (num_modes = 10) and an energy_range of 4; all other settings were left at their defaults. Generated poses were ranked according to the affinity scores produced by Vina’s internal scoring function. Protocol robustness was verified through redocking of the co-crystallized ligand: superposing the top-scoring predicted pose with the experimental conformation produced an RMSD of 0.316 Å, well below the widely accepted 2 Å threshold, thereby validating the reliability of the docking workflow. RMSD measurements were carried out in PyMOL 3.1.8 [35]. Finally, the top-ranked poses were visually inspected in ADT 1.5.7 and Discovery Studio to characterize key interactions (hydrogen bonds, hydrophobic contacts, etc.) and refine interpretation of the docking results.

### 2.4. Implementation of Molecular Dynamics Simulations Using GROMACS

The protein crystal structure (P.pdb) was first curated in UCSF Chimera 1.19 to delete extraneous hetero-molecules and resolve minor steric or connectivity issues. The resulting clean model was processed with gmx pdb2gmx (GROMACS 2021.4) using the CHARMM27 force field, which automatically appended any missing hydrogens and assigned the appropriate protonation states while generating the full topology. Each ligand was parameterised separately via the SwissParam web server, yielding CHARMM-compatible topology (.itp) and coordinate (.pdb) files. The ligand coordinates were converted to the GROMACS-ready .gro format and merged with the protein to create a single protein–ligand complex. The complex was centered in a cubic simulation box, solvated with TIP3P water, and electrically neutralized by adding counter-ions. Energy minimisation and double-stage equilibration (NVT followed by NPT) were then conducted under standard settings defined in the accompanying .mdp files [36]. Finally, a 100 ns production molecular dynamics simulation was carried out, during which atomic coordinates and velocities were periodically recorded. This approach facilitated an accurate assessment of the stability, structural dynamics, and critical ligand–protein interactions within each complex.

### 2.5. MM/GBSA Calculation

Binding free energies (ΔG_bind_) were computed by MM/GBSA in AmberTools23 (MMPBSA.py, parallel mode). A single-trajectory scheme sampled 100 snapshots at 0.25 ns intervals from the 75–100 ns window of the GROMACS simulation. This late window was selected because it follows the approximately 35 ns ligand reorientation and represents the final RMSD plateau; using the entire trajectory would mix the initial exploration, transition, and late adapted states. The HCT Generalized-Born model (igb = 5) was used with ε_in_ = 1.0, ε_out_ = 80.0, and an ionic strength of 0.15 M; the nonpolar term was derived from SASA. Per-residue decomposition (idecomp = 1) reported van der Waals, electrostatic, polar (GB), and nonpolar (surface) contributions. Temporary files were removed after completion (keepfiles = 0).

## 3. Results and Discussion

### 3.1. Physicochemical Attributes and Pharmacokinetic Parameters (ADME)

#### 3.1.1. Comparative Analysis of ADME Profiles and Drug-likeness Using SwissADME Radar Plots

**Figure 1:**
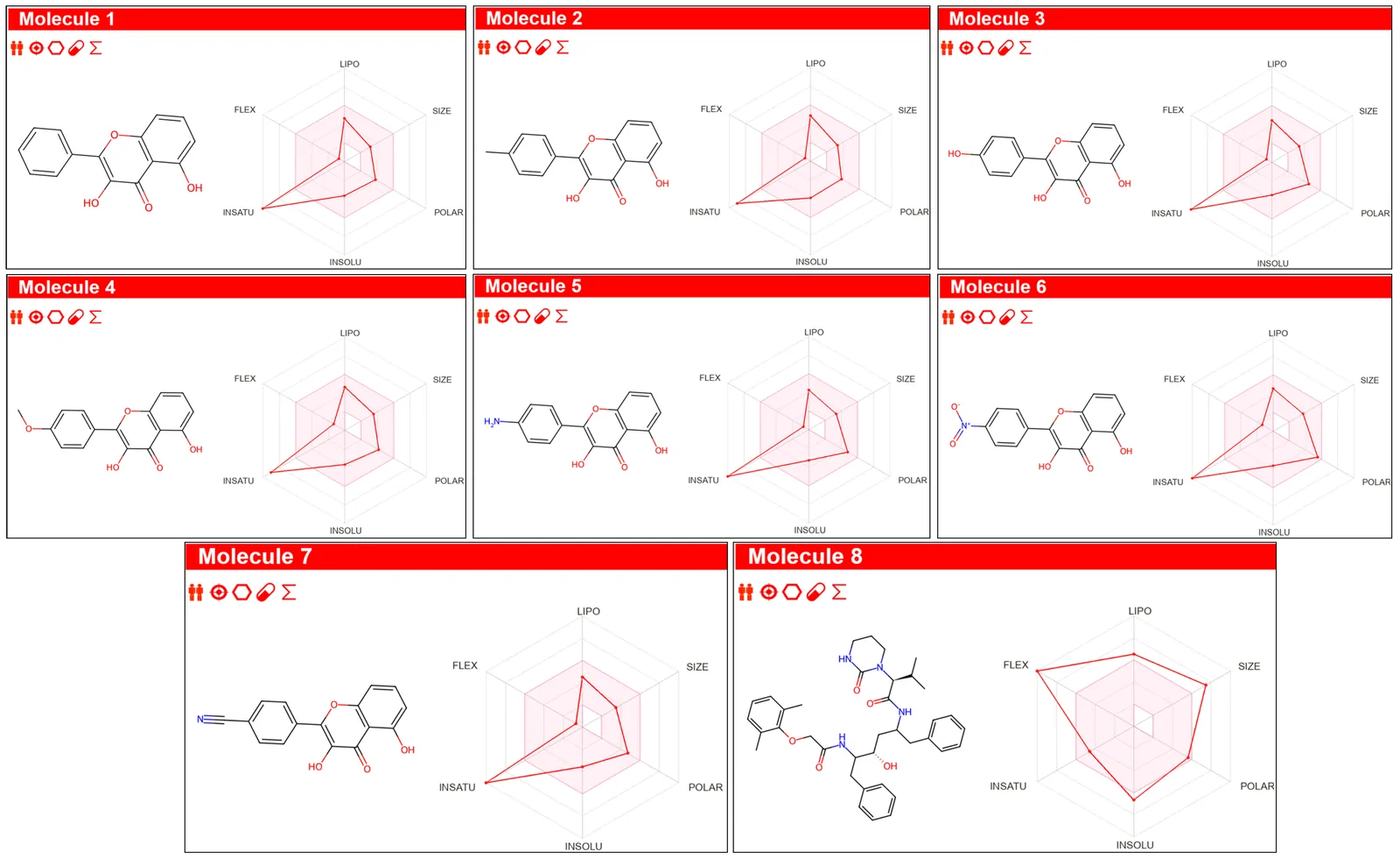
SwissADME radar charts: ADME profiles of the studied molecules.

From a pharmacokinetic and pharmacodynamic standpoint, the radar analysis generated by tools such as SwissADME (Figure 1) highlights a set of key parameters: lipophilicity, aqueous solubility, polarity (often assessed via total polar surface area), and the number of hydrogen-bond donors and acceptors that directly influence the therapeutic potential and metabolic stability of our seven compounds [28,37]. Without revealing specific numerical values, one can deduce that these compounds, mostly structured around aromatic rings and enriched with polar groups (hydroxyls, carbonyls, etc.), exhibit a mixed profile in terms of solubility and membrane permeability. The balance between polar sites and hydrophobic surfaces can be critical for intestinal absorption (and, thus, possible oral bioavailability), as well as for plasma protein binding and tissue distribution, important considerations given that the therapeutic target (e.g., the 3CL^pro viral protease or the polymerase) is located within infected cells. Lopinavir (M8) was retained here only as a historical antiviral and ADMET comparator, not as an M^pro^-specific positive control; its relatively large, lipophilic profile provides a contrasting reference for the designed series. For M1–M7, these radar descriptors are qualitative and do not establish more efficient cellular uptake or superior in vivo exposure; the hydroxylated analogues may also remain susceptible to phase-II biotransformation. Furthermore, possible interactions with certain cytochrome P450 isoforms or efflux transporters need to be considered to anticipate risks of rapid metabolism or low bioavailability.

#### 3.1.2. ADME Profiles of the Investigated Molecules Assessed via SwissADME and the “BOILED-EGG” Method

Table 1 summarizes the SwissADME physicochemical and rule-based drug-likeness descriptors for M1–M7 and lopinavir, providing a suitable basis for systematic comparison of the compounds and evaluation of their preliminary drug-likeness-related properties. Indeed, all seven compounds display a substantially lower molecular weight than 500 g/mol (ranging from 254 g/mol to 299 g/mol), thus meeting one of the key criteria of Lipinski’s “Rule of Five” without demonstrating measured oral absorption [38,39]. Additionally, their low number of rotatable bonds (ranging from 1 to 2) imparts greater conformational rigidity, which reduces conformational flexibility but does not by itself establish membrane permeability or bioavailability. These compounds also exhibit moderate cLogP values (approximately 1.6 to 2.7), favoring a satisfactory balance between lipophilicity and solubility. Furthermore, their topological polar surface areas (TPSAs) of around 70 Å^2^ to 116 Å^2^ fall below the critical 140 Å^2^ threshold recommended by Veber’s rule, satisfying the Veber TPSA criterion without establishing oral absorption [40]. Regarding Lipinski, Veber, and Ghose criteria, the seven compounds show no violations, supporting compliance with these rule-based drug-likeness filters. By contrast, lopinavir, despite having been clinically studied in other indications and presented here as a reference, reveals several significant drawbacks: its molecular weight (628.8 g/mol) considerably exceeds the Lipinski threshold of 500 g/mol, and it exhibits a very high number of rotatable bonds (17), increasing its structural flexibility and complicating its formulation often necessitating a pharmacokinetic “boost” (e.g., with ritonavir) to achieve adequate plasma concentrations. In addition, lopinavir violates three of Ghose’s criteria, reflecting excessive molecular bulk and a higher number of atoms than typically recommended for orally active small molecules. Thus, although lopinavir maintains a nominally acceptable bioavailability (SwissADME score of 0.55), its large and highly flexible structure complicates its use and may interfere with its distribution, metabolism, and excretion.

**Figure 2:**
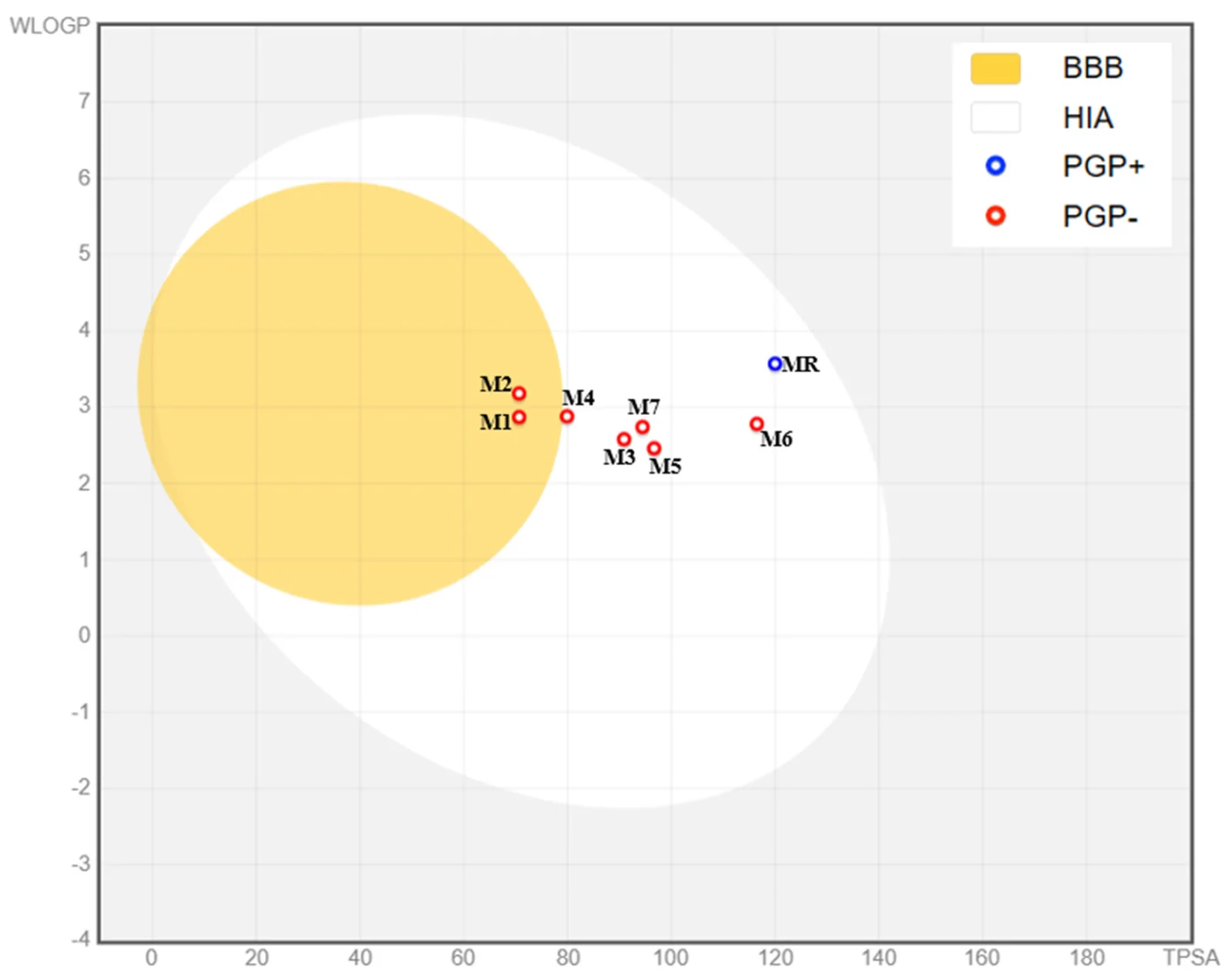
SwissADME “BOILED EGG”: summary of intestinal permeability, BBB access, P-gp interaction, and polarity/lipophilicity.

The “BOILED-EGG” diagram shown here is a predictive tool developed in particular by SwissADME (Figure 2) [41]. It allows visualization, on a single plot, of the potential for gastrointestinal absorption (HIA, in white), blood–brain barrier permeability (BBB, in yellow), and possible interactions with P-glycoprotein (P-gp). The x-axis (TPSA) represents the topological polar surface area, a parameter correlated with absorption and tissue permeation capacity, while the y-axis (WLOGP) indicates lipophilicity. The colored areas (yellow ellipse for BBB, white ellipse for HIA) suggest whether, according to these two parameters, a molecule is more likely to be well absorbed orally and/or to cross the blood–brain barrier. Furthermore, the colored data points convey the likelihood of interaction with P-gp: a “PGP+” compound (blue point) is likely to be a P-gp substrate (risk of efflux leading to decreased intracellular concentrations), whereas a “PGP-“ (red point) is less so. In this diagram, the seven compounds of interest (M1–M7) are all positioned in the white area or at the boundary between the white and yellow zones, indicating a favorable prediction of intestinal absorption (HIA) and, in some cases, a somewhat limited BBB permeability. Their distribution at TPSA values roughly between 60 and 100 Å^2^ and a WLOGP around 2 to 3 suggests a favorable compromise between polarity and lipophilicity. Within this model, M1, M2, M3, M4, M5, and M7 are classified as P-gp non-substrates (red points); this classification does not measure bioavailability or exclude transporter-mediated drug–drug interactions. M6 occupies a somewhat more lipophilic position while remaining within the model’s gastrointestinal-absorption domain.

The reference molecule (MR) here, corresponding to lopinavir (blue point), lies in the white zone but shows a slightly higher TPSA and a higher WLOGP (around 4), classifying it as “PGP+.” The model therefore classifies lopinavir as a P-gp substrate, a result that flags possible efflux and transporter-mediated interaction liabilities but is not a measured pharmacokinetic outcome. In practice, lopinavir is frequently combined with ritonavir to partly circumvent these issues, highlighting its major disadvantage: the need for a “booster” (ritonavir) to achieve adequate plasma levels, which in turn increases the risk of adverse effects and interactions. The relative positions of M1–M7 and lopinavir in the BOILED-EGG plot support model-based HIA and P-gp-substrate comparisons only; they do not demonstrate superior pharmacokinetics or reduced administration constraints.

#### 3.1.3. Comprehensive Assessment of ADMET Profiles and Their Significance in SARS-CoV-2 Therapeutic Development

Based on the ADMETlab 3.0 data provided in Table 2, a thorough comparison of the seven new compounds against the reference compound lopinavir, in the context of potential SARS-CoV-2 inhibitors, can be drawn by considering several key parameters: human intestinal absorption (HIA), Caco-2 permeability, MDCK permeability, and PAMPA diffusion. First, the HIA entries are classification probabilities reported as percentages: M1, M2, M3, M5, and M6 range from 0.79% to 2.5%, whereas M4 and M7 show values of 20% and 37%, respectively. Lopinavir has an HIA-positive probability of 0.0242%. These values should be interpreted strictly as model-derived classification probabilities and not as direct indicators of oral absorption or bioavailability. The Caco-2 and MDCK entries are regression estimates expressed as log10(Papp, cm s^−1^), not measured permeability values. Within these model outputs, M1 and M2 have less negative Caco-2 estimates than lopinavir [42,43], while M1 and M6 show the least negative MDCK estimates among the M1–M7 series. PAMPA is reported as a classification probability for high permeability, with M6 displaying the highest value among the designed compounds (0.83), compared with 0.003 for lopinavir, thereby distinguishing M6 as the most favorable candidate in this endpoint. The transporter profile further reveals that M1, M2, M4, M6, and M7 have high predicted probabilities of P-gp inhibition, whereas most members of the M1–M7 series show low probabilities of being P-gp substrates. This pattern may favor intracellular retention for selected compounds, although strong P-gp inhibition also raises the possibility of transporter-mediated drug–drug interactions. Beyond P-gp, the uniformly high predicted probabilities of OATP1B1 and OATP1B3 inhibition across M1–M7 indicate a relevant potential for interactions involving hepatic uptake transporters [44]. The predicted inhibition profiles of BCRP, BSEP, and MRP1 are more heterogeneous, highlighting compound-specific differences in transporter liability across the series [45]. Distribution-related predictions show consistently high plasma protein binding, with PPB values exceeding 97%, together with low BBB-positive probabilities and negative logVDss estimates, suggesting extensive protein association, limited predicted CNS penetration, and relatively restricted systemic distribution volumes. Lopinavir falls within a comparable predicted logVDss range but differs substantially from the designed compounds in several permeability and transporter-related endpoints. Taken together, these results define distinct ADMET profiles within the M1–M7 series and identify M6, in particular, as noteworthy for its PAMPA and MDCK characteristics; however, the entire dataset should be regarded as a model-based screening framework that requires experimental validation of permeability, transporter interactions, protein binding, and in vivo pharmacokinetic behavior before any definitive pharmacokinetic advantage can be established.

#### 3.1.4. Comparative Evaluation of Inhibition Profiles and Pharmacokinetic Parameters

Based on Table 3, a combined analysis of their inhibition and substrate probabilities with respect to the major CYP450 enzymes, along with key pharmacokinetic parameters (plasma clearance and half-life), reveals several notable differences that may influence their drug interaction profiles and metabolic fates. M1–M7 differ from lopinavir by showing a high likelihood of being both a substrate and an inhibitor of CYP2C9, whereas lopinavir exhibits virtually no involvement with this pathway. Similarly, M1–M3 display strong inhibitory potential toward CYP1A2, while lopinavir barely inhibits it at all. Regarding CYP2C19, lopinavir stands out as significantly more likely to be a substrate (0.91) than most of the new candidates (≤0.004), whereas for CYP2D6, all the tested compounds show a very low inhibition risk combined with a substantial probability of being metabolized by this enzyme, in contrast to lopinavir (0.012) [46]. Furthermore, CYP3A4, a major determinant of drug–drug interactions, is predicted to involve lopinavir as both inhibitor (0.98) and substrate (0.97), whereas the lower inhibitor probabilities of M1–M7 require experimental confirmation and do not exclude interactions through other CYP or transporter pathways.

**Figure 3:**
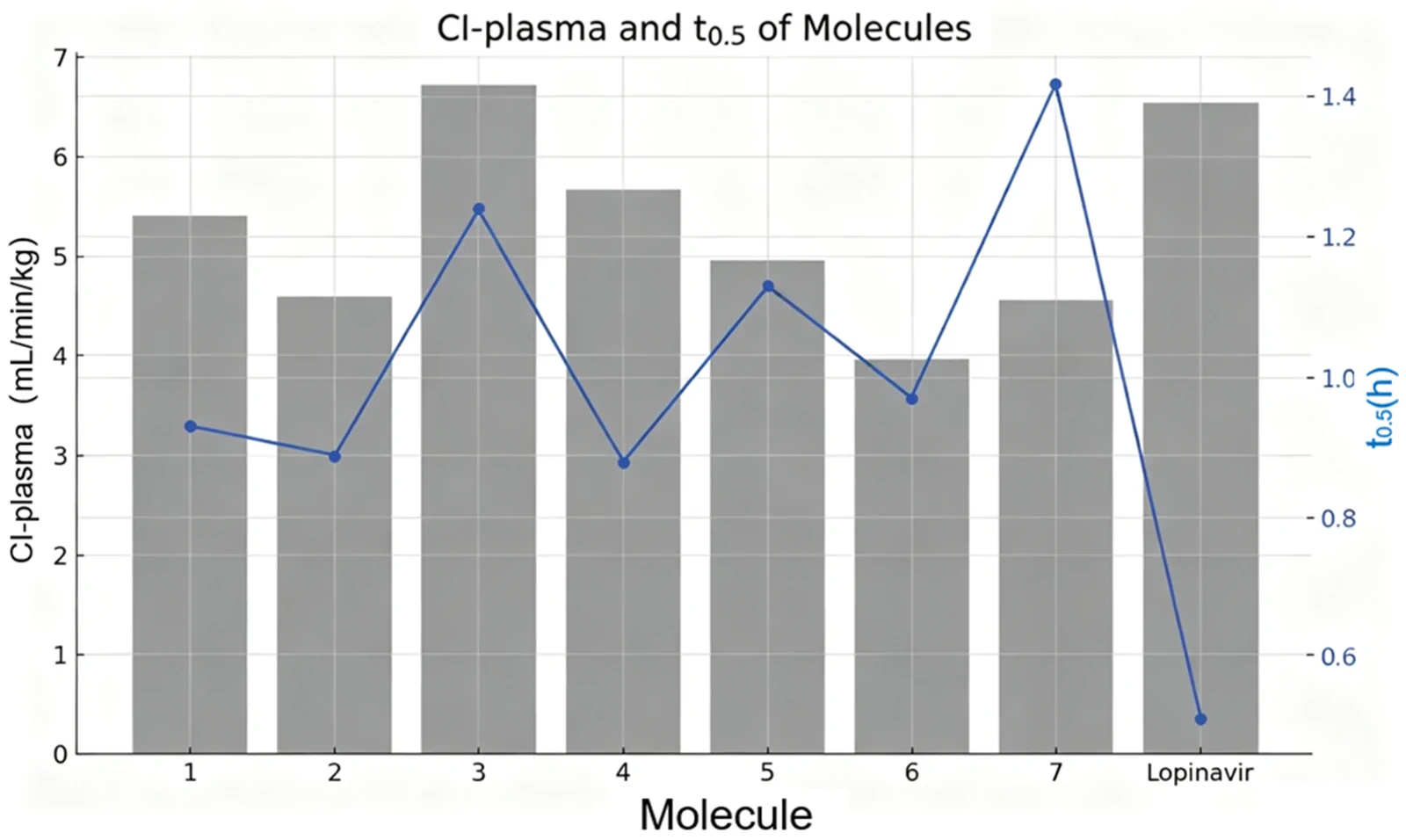
Comparison of plasma clearance (Cl-plasma) and half-life (t_0.5_) for the seven compounds and lopinavir.

The data presented in Figure 3 provide a detailed overview of two essential pharmacokinetic parameters: plasma clearance (cl-plasma) and half-life (t_0_._5_). Plasma clearance reflects how quickly the body eliminates a molecule (via the liver, kidneys, or other metabolic pathways), whereas half-life indicates how long the compound remains in the body before its concentration is reduced by half. A compound with a lower plasma clearance is typically removed more slowly, which often corresponds to a longer half-life and, consequently, an extended residence time in the bloodstream. Within the ADMETlab model, M7 has a lower predicted plasma clearance (4.56 mL min^−1^ kg^−1^) and a longer predicted half-life (1.42 h) than lopinavir (6.55 mL min^−1^ kg^−1^ and 0.51 h, respectively). These estimates distinguish M7 within the series but do not establish slower in vivo elimination, reduced dosing frequency, or a wider therapeutic window; accumulation, efficacy, and safety require experimental pharmacokinetic evaluation.

#### 3.1.5. Multi-Target Binding Profiles and Their Relevance for SARS-CoV-2 Inhibition

**Figure 4:**
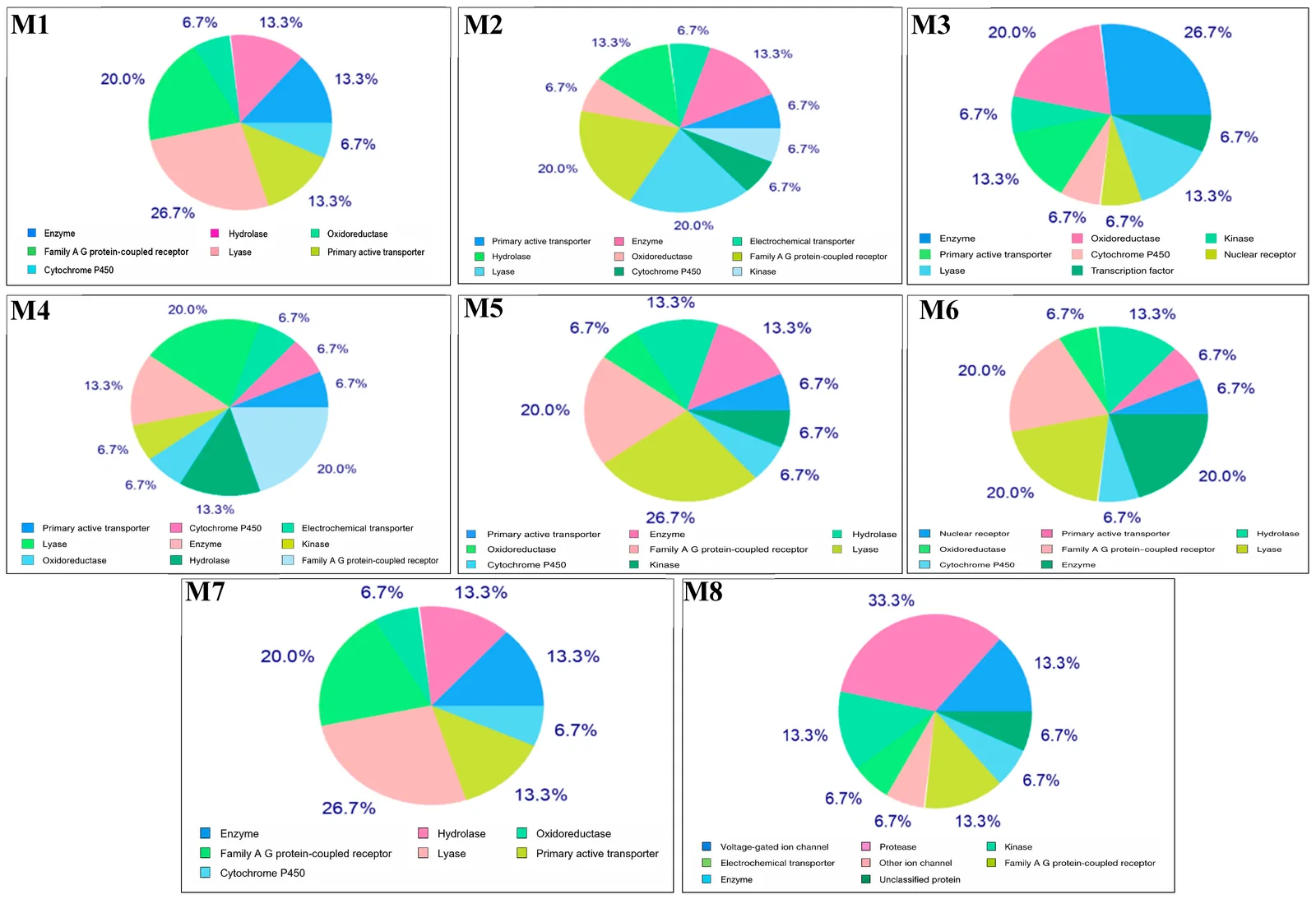
SwissADME-predicted protein target profiles for eight compounds.

The profiles in Figure 4 show that the predicted binding profiles are predominantly distributed among several protein target families: enzymes (including proteases and hydrolases), transporters (particularly primary active transporters), and G protein-coupled receptors (class A GPCRs) [47]. For compounds M1–M7, the target-family distributions generated by SwissTargetPrediction serve as a hypothesis-generating framework for potential molecular targets and suggest possible associations with hydrolases, oxidoreductases, transporters, and G protein-coupled receptors (GPCRs). The reference compound M8 (lopinavir) shows a substantial proportion of predicted interactions with proteases, consistent with its established protease-inhibitor activity in antiretroviral therapy. Predictions involving kinases, ion channels, and other target classes further suggest a broader spectrum of potential off-target interactions. Overall, SwissTargetPrediction provides a useful predictive map of potential molecular targets that can help prioritize the most relevant compounds and pathways for experimental investigation, including interaction with SARS-CoV-2 M^pro^.

### 3.2. ProTox-III Toxicity Radar

**Figure 5:**
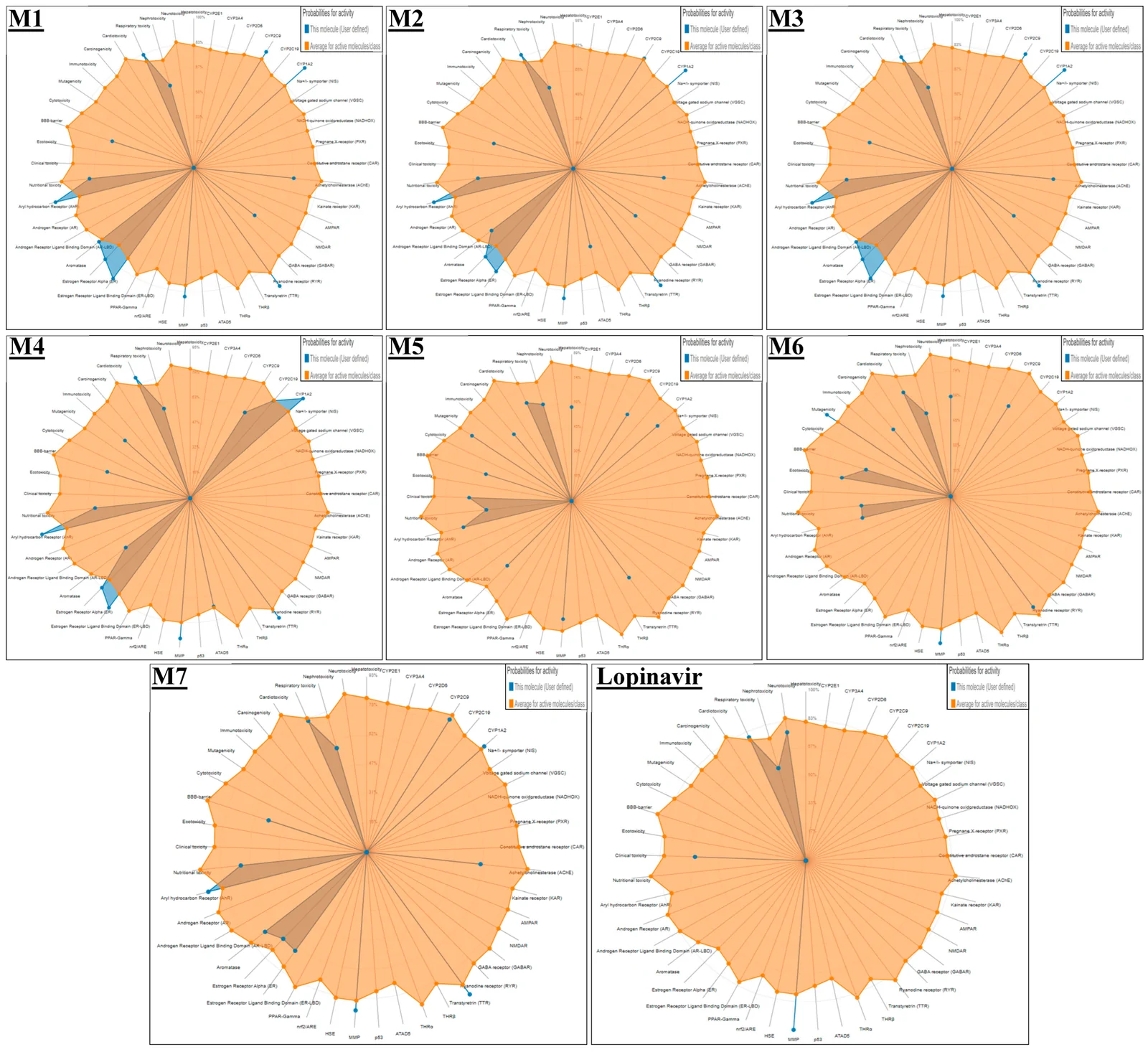
ProTox-III toxicity radar profiles of M1–M7 and lopinavir. Blue points/lines show the predicted probability for each query compound, whereas orange points/lines show the mean probability of active compounds in the corresponding model training set.

The ProTox-III radar results for these seven structurally related compounds, each bearing a single functional group modification relative to a common core scaffold, reveal marked variations in predicted toxicity profiles and underscore how subtle structural changes can profoundly influence toxicological behavior (Figure 5) [30]. M1 shows high query probabilities for respiratory toxicity, CYP1A2/CYP2C9-related endpoints, and several endocrine-receptor models, whereas lower values for nephrotoxicity, BBB, AChE, and GABAR are simply lower model probabilities. The orange profile represents the mean probability of active compounds in each training set and is not a universal toxicity threshold; therefore, values below it are not classified as safe. Relative to M1, M2 and M4 show lower predicted aromatase probabilities, while M4 also shows ATAD5 and immunotoxicity signals. M5 introduces predicted hepatotoxicity, clinical-toxicity, mutagenicity, and carcinogenicity liabilities, and M6 carries the strongest mutagenicity signal (97%) together with an ecotoxicity signal. M7 shows lower predicted aromatase, ER, and ER-LBD probabilities than M1, but these remain confidence estimates rather than incidence rates. Within the same model, lopinavir provides only a comparative prediction profile; its clinical adverse-effect record is not used to validate ProTox-III. Accordingly, M6 requires targeted Ames, micronucleus, cytotoxicity, hepatotoxicity, and off-target testing before biological development.

### 3.3. Molecular Docking

The docking results (Table 4), performed on the main protease (M^pro^) of SARS-CoV-2 (PDB code 9C8Q), which features critical catalytic and binding residues such as Cys145 and His41, highlight the importance of the substituent in modulating the binding affinity of the flavonoid derivatives M1–M7 toward this key enzyme [48]. Lopinavir was retained only as a historical antiviral and ADMET comparator, not as an M^pro^-specific positive control. The target-specific benchmark is the 9C8Q co-crystallized ligand, whose redocked pose reproduced the experimental conformation (RMSD 0.316 Å) and yielded a Vina score of −8.0 kcal mol^−1^, close to M6 (−8.1 kcal mol^−1^). M6 was the best-ranked designed analogue, with predicted contacts involving His41, Cys44, Phe140, His163, and Met165; nevertheless, lopinavir had a more negative Vina score (−9.2 kcal mol^−1^). M4, M3, and M5 gave closely grouped scores (−7.6 to −7.7 kcal mol^−1^) with distinct polar-contact patterns, whereas M1, M2, and M7 ranked between −7.2 and −7.5 kcal mol^−1^ [49]. These model-dependent scores do not establish experimental potency, and selected favorable ADMET predictions for M6 do not demonstrate compensation for its less negative score than lopinavir; biochemical M^pro^ inhibition and pharmacokinetic studies are required.

M3, M5, and M6 were selected for detailed interaction and DFT analyses as a mechanistically contrasting subset representing hydroxyl, amino, and nitro para substitution. The selection integrated substituent class, polar-contact topology, electronic contrast, and toxicity signals rather than docking score alone. M4 had a slightly better docking score than M3/M5, and M7 showed the highest HIA-positive probability; both are therefore acknowledged as relevant alternatives, but they were not included in the focused downstream subset because the objective was to compare three chemically distinct electronic regimes within the available computational scope.

**Figure 6:**
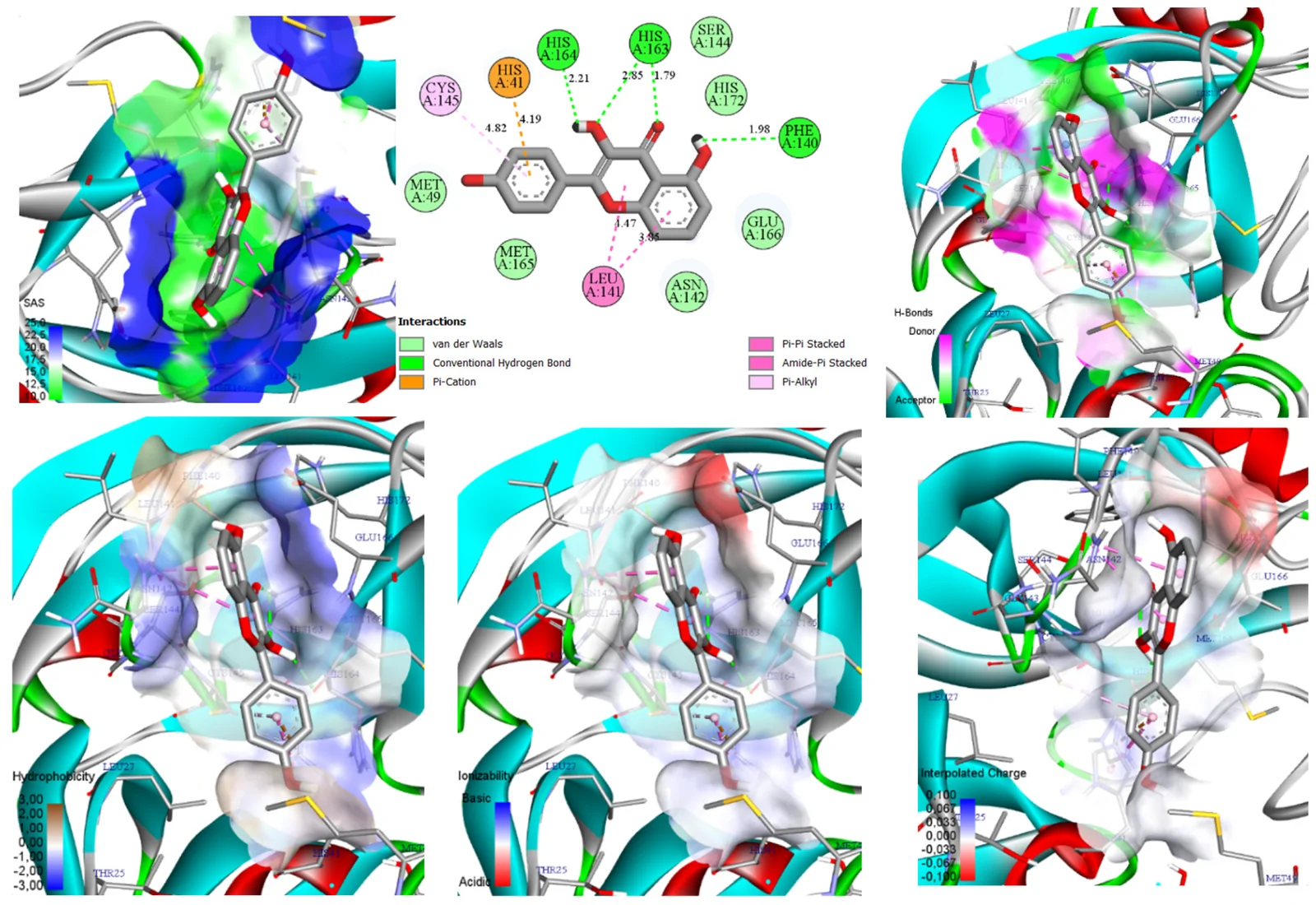
The 2D and 3D interactions of M3 with SARS-CoV-2 M^pro^. In the 2D map, light green denotes van der Waals contacts, bright green conventional hydrogen bonds, magenta π-π/amide-π contacts, and pale pink π-alkyl contacts; the 3D panels show solvent-accessible surface, hydrogen-bond donor/acceptor, hydrophobicity, ionizability, and interpolated-charge surfaces.

M3 showed a Vina score of −7.6 kcal mol^−1^ and a docked pose located in the M^pro^ catalytic pocket near the His41-Cys145 dyad (Figure 6). The 2D evaluation reveals exceptionally robust hydrogen bonding with His163 (2.85 Å and 1.79 Å) and His164 (2.21 Å), as well as a conventional hydrogen bond with Phe140 (1.98 Å) [50]. Complementary hydrophobic π-π interactions with Leu141 (3.85 Å and 4.47 Å), together with six van der Waals contacts, further consolidate the ligand’s stabilization within the cavity. The proximity to His41 (4.19 Å) and Cys145 (4.82 Å) identifies a predicted active-site geometry but does not establish catalytic modulation [51].

The 3D analysis, notably via the solvent-accessible surface (SAS) assessment, indicates that the molecule is effectively shielded from the external environment, thereby minimizing solvent exposure and enhancing interaction stability. An abundance of hydrogen bond donors and acceptors underscores the critical role of polar interactions, while the delineation of hydrophobic regions (illustrated in brown) mitigates energetic fluctuations and reinforces the ligand’s anchoring within the enzymatic pocket [52]. Furthermore, the ligand’s slightly basic ionization state promotes favorable electrostatic interactions with acidic or neutral active-site residues, and the interpolated charge map confirms an excellent electrostatic complementarity between molecule 3 and the catalytic cavity. Collectively, these contacts describe the predicted M3 docking pose but do not establish biochemical M^pro^ inhibition.

**Figure 7:**
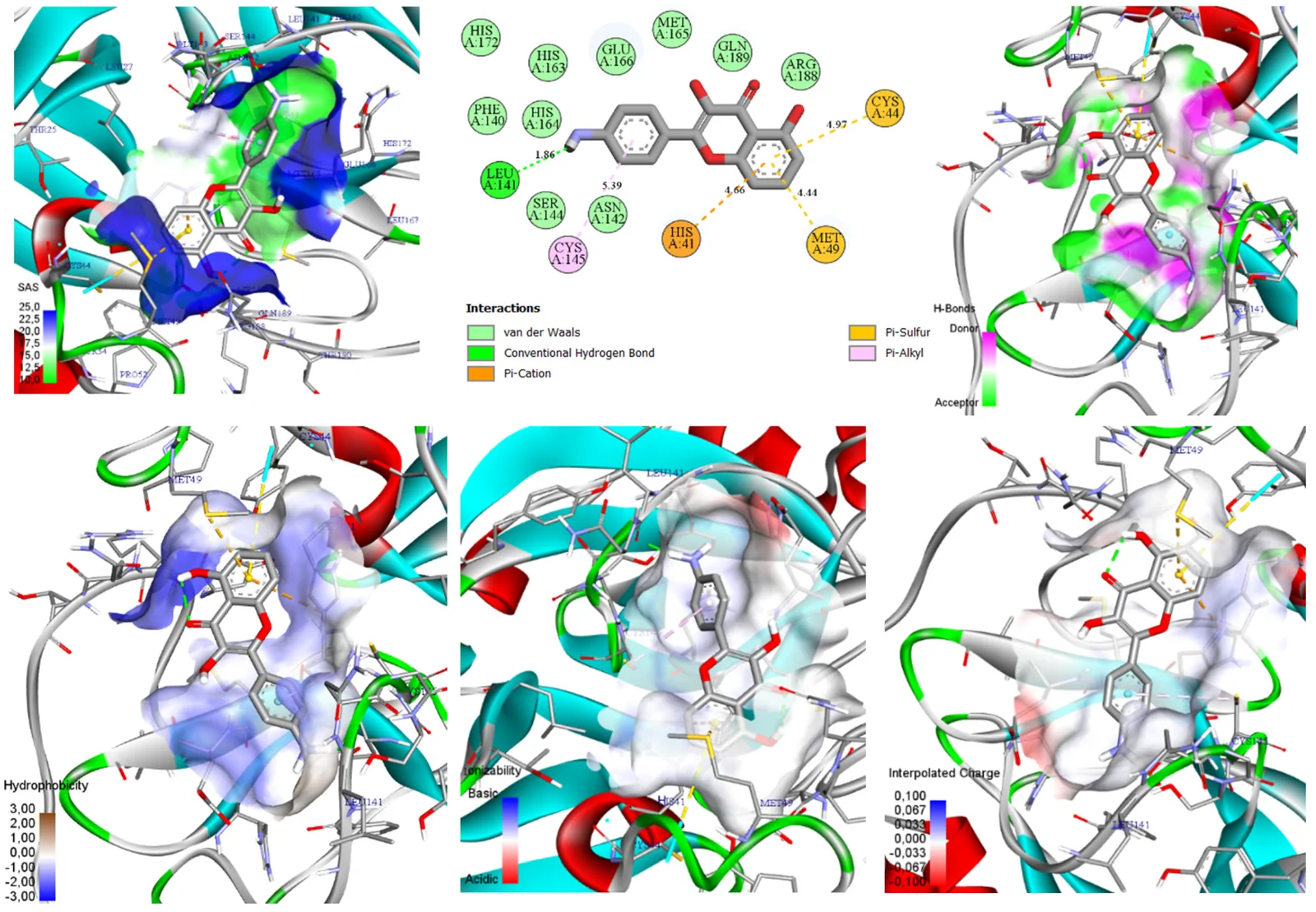
The 2D and 3D interaction analysis of M5 with SARS-CoV-2 M^pro^. Light green denotes van der Waals contacts, bright green conventional hydrogen bonds, orange π-cation contacts, yellow π-sulfur contacts, and pink π-alkyl contacts; the 3D panels show solvent-accessible surface, hydrogen-bond donor/acceptor, hydrophobicity, ionizability, and interpolated-charge surfaces.

M5 yielded a Vina score of −7.6 kcal mol^−1^ for the SARS-CoV-2 main protease. The corresponding docked pose displays the contacts visualized in the 2D and 3D analyses (Figure 7). In particular, the 2D analysis reveals a robust conventional hydrogen bond with residue Leu141 (1.86 Å), a stabilizing π-alkyl interaction with Cys145 (5.39 Å), and a noteworthy π-cation interaction with His41 (4.66 Å). Several π-sulfur interactions involving Cys44 (4.97 Å), Met49 (4.44 Å), and His41 further complement and strengthen the ligand’s specificity and electrostatic complementarity within the catalytic pocket. These specific interactions, together with ten van der Waals contacts involving His172, His163, Glu166, Met165, Gln189, Arg188, Phe140, His164, Ser144, and Asn142, perfectly account for the results observed in 3D analysis [53]. Indeed, the solvent-accessible surface (SAS) study demonstrates that the molecule is highly protected within the active site, minimizing its exposure to the solvent and further stabilizing the protein–ligand complex. The balanced distribution of hydrogen-bond donor and acceptor regions observed in the 3D analysis supports the polar interactions noted in 2D, while the complementarity of hydrophobic regions reduces energy fluctuations and aids ligand anchoring. The interpolated charge map and the slightly basic character of the ligand, as shown by ionizability analysis, confirm the importance of the electrostatic interactions highlighted by the π-cation and π-sulfur contacts. Moreover, an intramolecular CO-OH interaction significantly restricts the ligand’s conformational flexibility, thereby favoring its optimal orientation within the active site.

**Figure 8:**
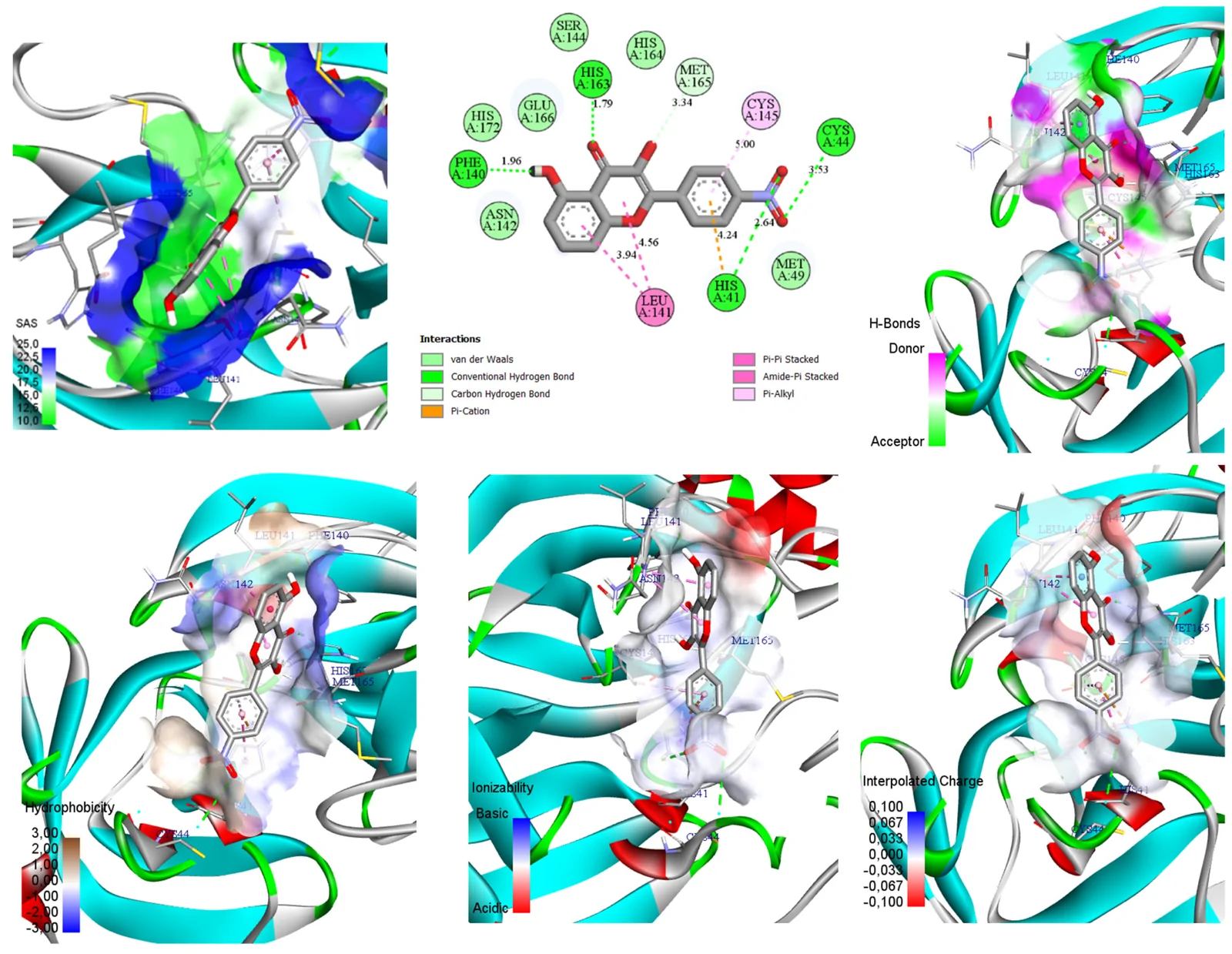
The 2D and 3D interaction analysis of M6 with SARS-CoV-2 M^pro^. Light green denotes van der Waals contacts, bright green conventional hydrogen bonds, pale green carbon–hydrogen bonds, orange π-cation contacts, magenta π-π/amide-π contacts, and pink π-alkyl contacts; the 3D panels show solvent-accessible surface, hydrogen-bond donor/acceptor, hydrophobicity, ionizability, and interpolated-charge surfaces.

M6 gave the best docking score among the designed analogues (−8.1 kcal mol^−1^), supported by the interaction pattern shown in the 2D and 3D analyses (Figure 8). In particular, the molecule establishes strong conventional hydrogen bonds with the critical residues His163 (1.79 Å), Phe140 (1.96 Å), and Cys44 (3.53 Å), as well as a notably significant bond with His41 (2.64 Å), promoting robust and specific anchoring in the catalytic pocket. A predicted π-cation contact with His41 (4.24 Å), a residue of the His41-Cys145 catalytic dyad, places M6 near the catalytic environment without demonstrating enzymatic modulation. Additional π-alkyl, π-π/amide-π, and carbon–hydrogen contacts with Cys145, Leu141, and Met165 define the initial docked geometry [54,55]. The 3D surfaces visualize solvent exposure, donor/acceptor regions, hydrophobicity, ionizability, and interpolated charge for this static pose; they do not independently establish dynamic stability. The complementarity of hydrogen-bond donors and acceptors, the precise alignment of hydrophobic regions, and the optimal electrostatic compatibility visible in the interpolated charge and ionizability maps all reinforce the findings of the 2D analysis. The docking poses and comparative ADMET outputs support prioritization of M6 for testing, but its predicted mutagenicity signal and the absence of experimental validation preclude a therapeutic-efficacy or safety conclusion.

### 3.4. Density Functional Theory (DFT) Study

#### 3.4.1. Molecular Orbitals Analysis

**Figure 9:**
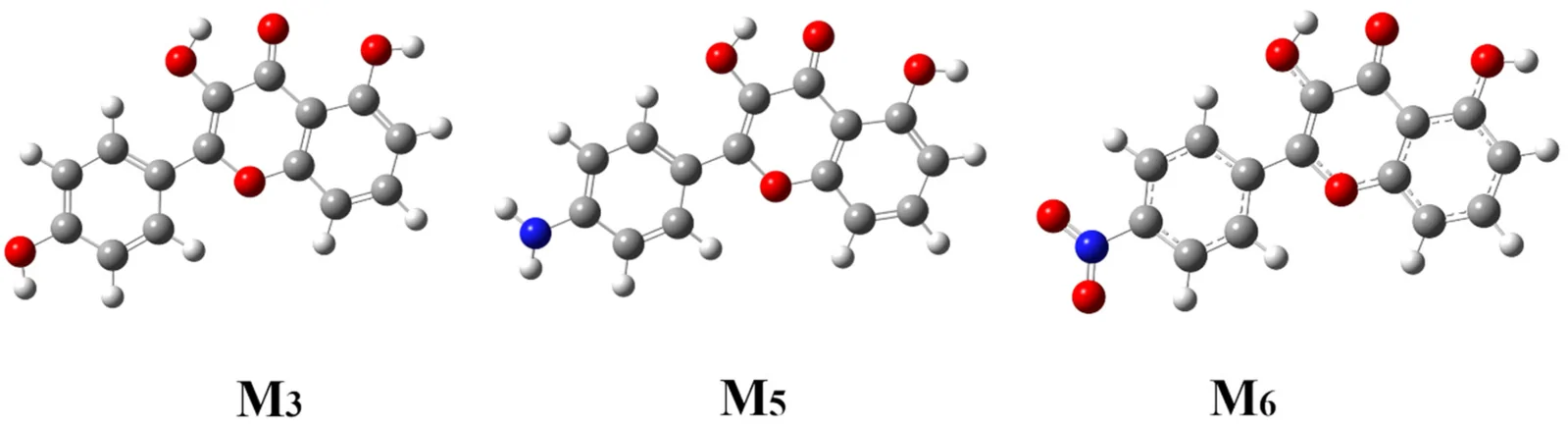
Optimized structures of M_3_, M_5_ and M_6_.

**Figure 10:**
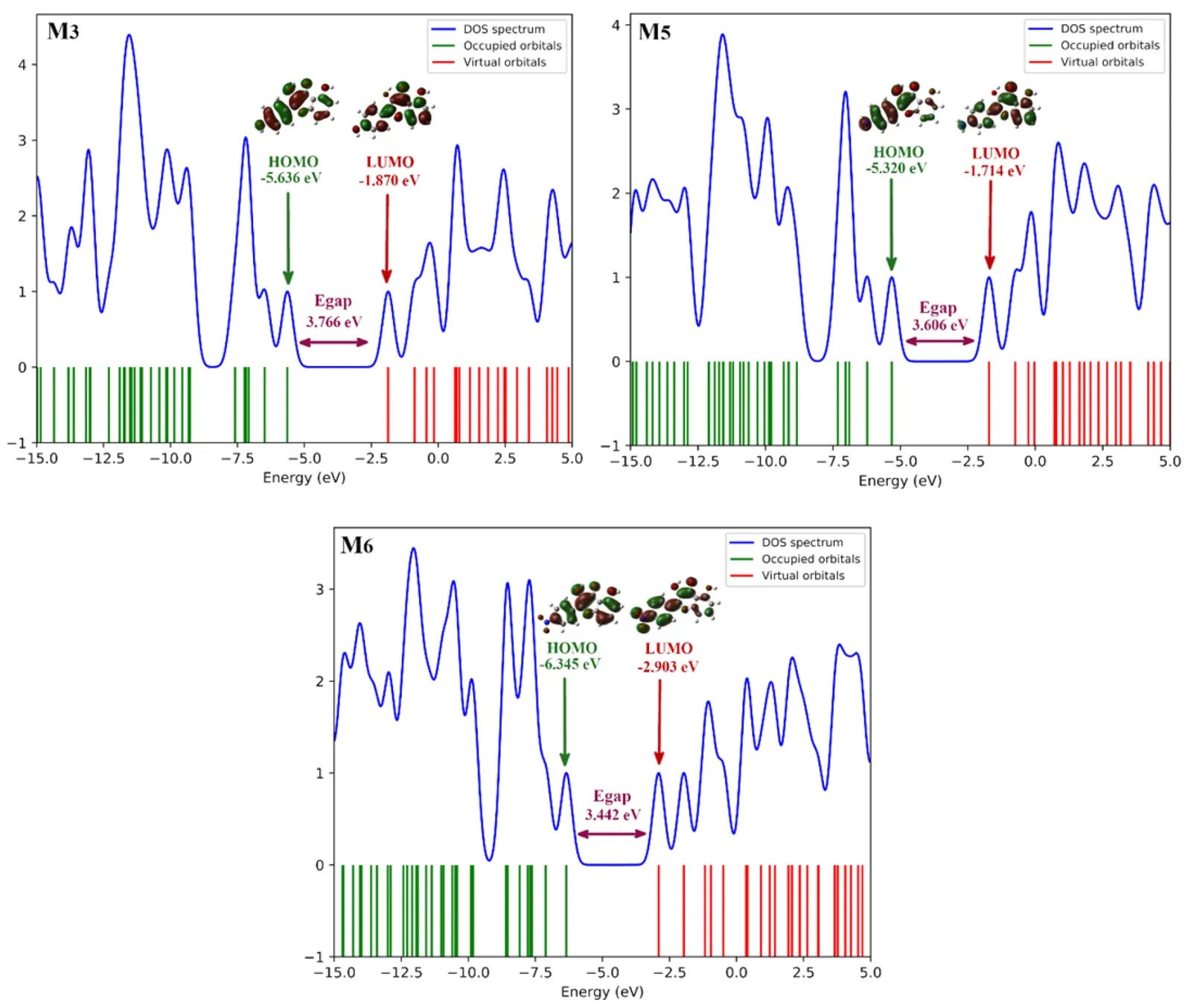
DOS Plot and FMOs of studied compounds M_3_, M_5_ and M_6_.

The optimized configurations of compounds M_3_, M_5_ and M_6_ are illustrated in Figure 9. Their stability is confirmed by the absence of imaginary frequencies. The electronic properties and reactivity of the studied compounds were investigated using frontier molecular orbital (FMO) analysis and density of states (DOS) calculations, providing insight into their chemical stability and interaction potential. The HOMO-LUMO energy levels play a crucial role in determining the electronic behavior of a system, as they dictate the molecule’s ability to donate or accept electrons. The HOMO represents the molecule’s nucleophilic character, while the LUMO signifies its electrophilic nature. A smaller HOMO-LUMO gap generally correlates with enhanced chemical reactivity, whereas a larger gap indicates increased kinetic stability and lower reactivity [56,57,58]. To analyze these electronic properties, quantum chemical calculations (DFT) were performed using Gaussian 09 software at the B3LYP/6-311G(d,p) level of theory [59,60], with the exact-exchange and LYP correlation components explicitly considered [61,62,63]. This provides reliable estimations of molecular orbitals and electronic distribution (Figure 10). No empirical dispersion correction was applied. Because these calculations concern isolated ligand geometries rather than the noncovalent protein–ligand complex, omission of D3 is expected to affect absolute dispersion-sensitive conformational energies more than the qualitative orbital ordering; a compound-specific numerical error cannot be assigned without paired B3LYP-D3(BJ) calculations [64]. From Table 5, it is clear that the computed HOMO-LUMO energy gaps indicate that M_6_ exhibits the lowest gap (3.442 eV), followed by M_5_ (3.606 eV) and M_3_ (3.766 eV). This result suggests that M_6_ is more chemically active than M_5_ and M_3_. Furthermore, M_5_ with a higher HOMO energy (−5.320 eV) shows a stronger tendency to donate electrons, making it a better nucleophile, while M_6_ with a lower LUMO energy (−2.903 eV) is more electrophilic and prone to electron-accepting interactions. The DOS analysis further supports these findings, illustrating the electronic state distribution and shedding light on potential charge transfer processes. Additionally, the spatial mapping of HOMO and LUMO orbitals highlights key reactive regions within each molecule, providing a deeper understanding of their molecular interactions and electronic transitions. These computational results confirm that M_6_ is the most reactive species due to its smaller gap and enhanced electrophilic nature. These FMO and global reactivity descriptors characterize isolated molecules and do not directly quantify protein–ligand binding affinity. Establishing residue-specific charge transfer or a causal link between electrophilicity and M^pro^ binding would require dedicated QM/MM or energy-decomposition analyses.

#### 3.4.2. Molecular Electrostatic Potential Investigation

**Figure 11:**
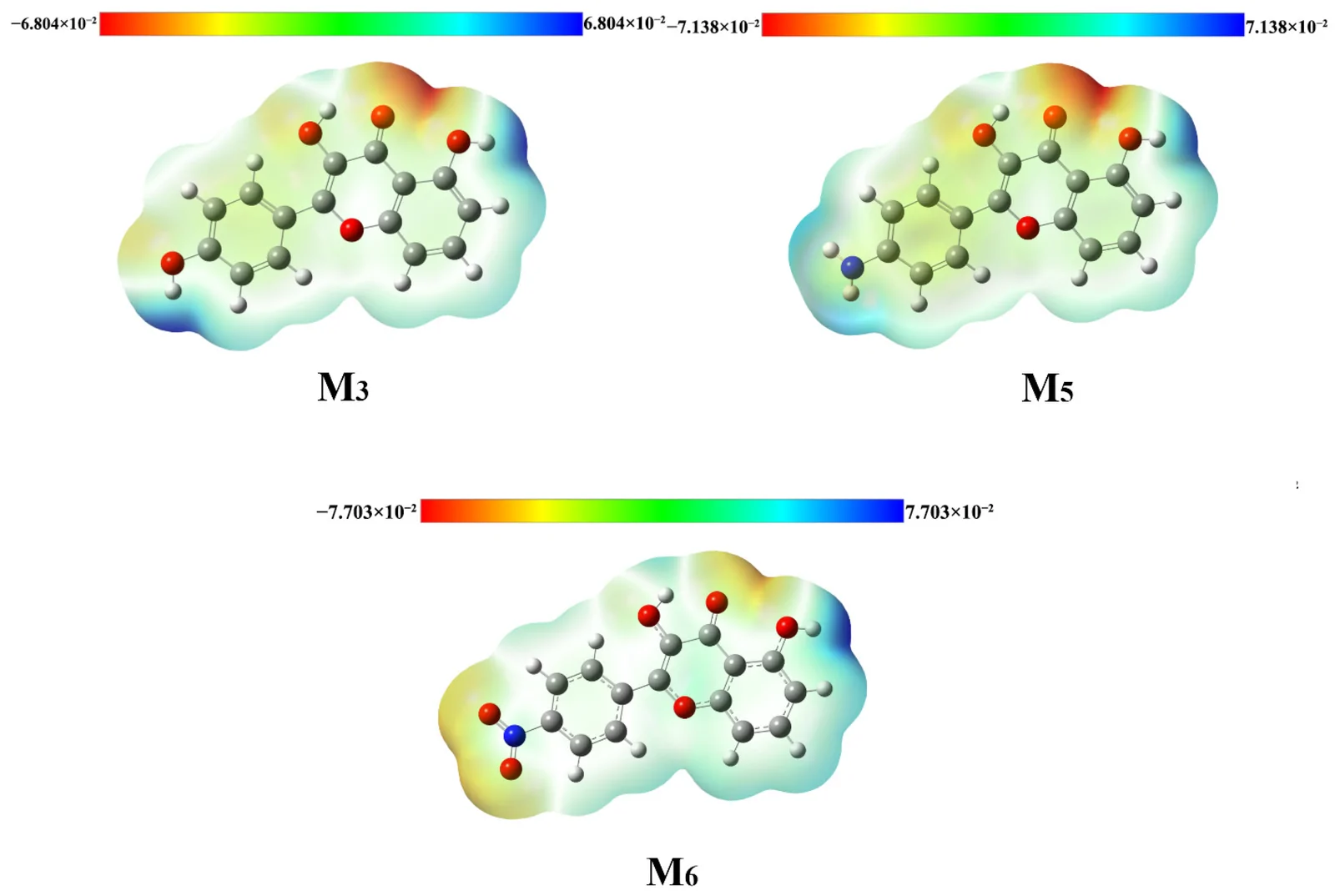
Molecular electrostatic potential of M_3_, M_5_ and M_6_.

The molecular electrostatic potential (MEP) maps (Figure 11) provide a detailed visualization of the charge distribution and potentially reactive sites [65] within the studied compounds (M_3_, M_5_ and M_6_). The color gradient, spanning from red (high electron density, negative potential) to blue (electron-deficient, positive potential), highlights zones susceptible to electrophilic and nucleophilic interactions. In all three compounds, the most electron-rich regions (red) are localized around the oxygen atoms of the carbonyl units, indicating strong sites for electrophilic attack. Conversely, electron-deficient regions (blue) are mainly distributed around hydrogen atoms linked to oxygen and nitrogen atoms, suggesting favorable sites for nucleophilic interactions. A comparative analysis reveals that M_6_ exhibits the most pronounced charge separation, with intensified red regions near oxygen atoms and enhanced blue regions around electropositive sites. This greater charge polarization suggests that M_6_ is the most chemically reactive compound, aligning with its lower HOMO-LUMO energy gap. M_5_, while sharing similar features with M_3_, presents a slightly increased electropositive region near the nitrogen atom, indicating modified electronic properties. These findings, corroborated by the HOMO-LUMO and DOS analysis, confirm that M_6_ is the most reactive molecule.

#### 3.4.3. Chemical Reactivity Parameters

We determined key chemical reactivity descriptors using the equations below, including chemical potential (μ), electronegativity (χ), electrophilicity (ω), softness (S) and chemical hardness (η) [66,67]. These calculations offer valuable insights into the stability, reactivity and potential biological interactions of the drug compounds M_3_, M_5_ and M_6_.

(1) $$\mu =\frac{\left(E_{HOMO}+E_{LUMO}\right)}{2}$$

(2) $$\chi =-\frac{\left(E_{HOMO}+E_{LUMO}\right)}{2}$$

(3) $$\eta =\frac{\left(E_{LUMO}-E_{HOMO}\right)}{2}$$

(4) $$\omega =\frac{\mu^{2}}{2\eta }$$

(5) $$S=\frac{1}{2\eta }$$

From Table 6, it is evident that among those studied compounds, M_6_ exhibits the highest electronegativity (χ = 4.624 eV), indicating a stronger tendency to attract electrons. Furthermore, its chemical hardness (η = 1.721 eV) is the lowest in the series, making M_6_ the softest and most reactive species. This observation is further supported by its high electrophilicity index (ω = 6.21 eV)ω, suggesting a strong ability to accept electrons, which may influence its interactions with nucleophiles in biological environments. Moreover, M_3_ and M_5_ exhibit intermediate values of μ, χ and η, implying moderate reactivity and a lower tendency for electron acceptance compared to M_6_. These descriptors do not demonstrate stronger biological-target binding, pharmacological efficacy, pharmacokinetic stability, or lower toxicity. Any residue-specific electronic interaction would require explicit treatment of the protein environment, and the biological implications must be established experimentally.

#### 3.4.4. Reduced Density Gradient (RDG) Study

**Figure 12:**
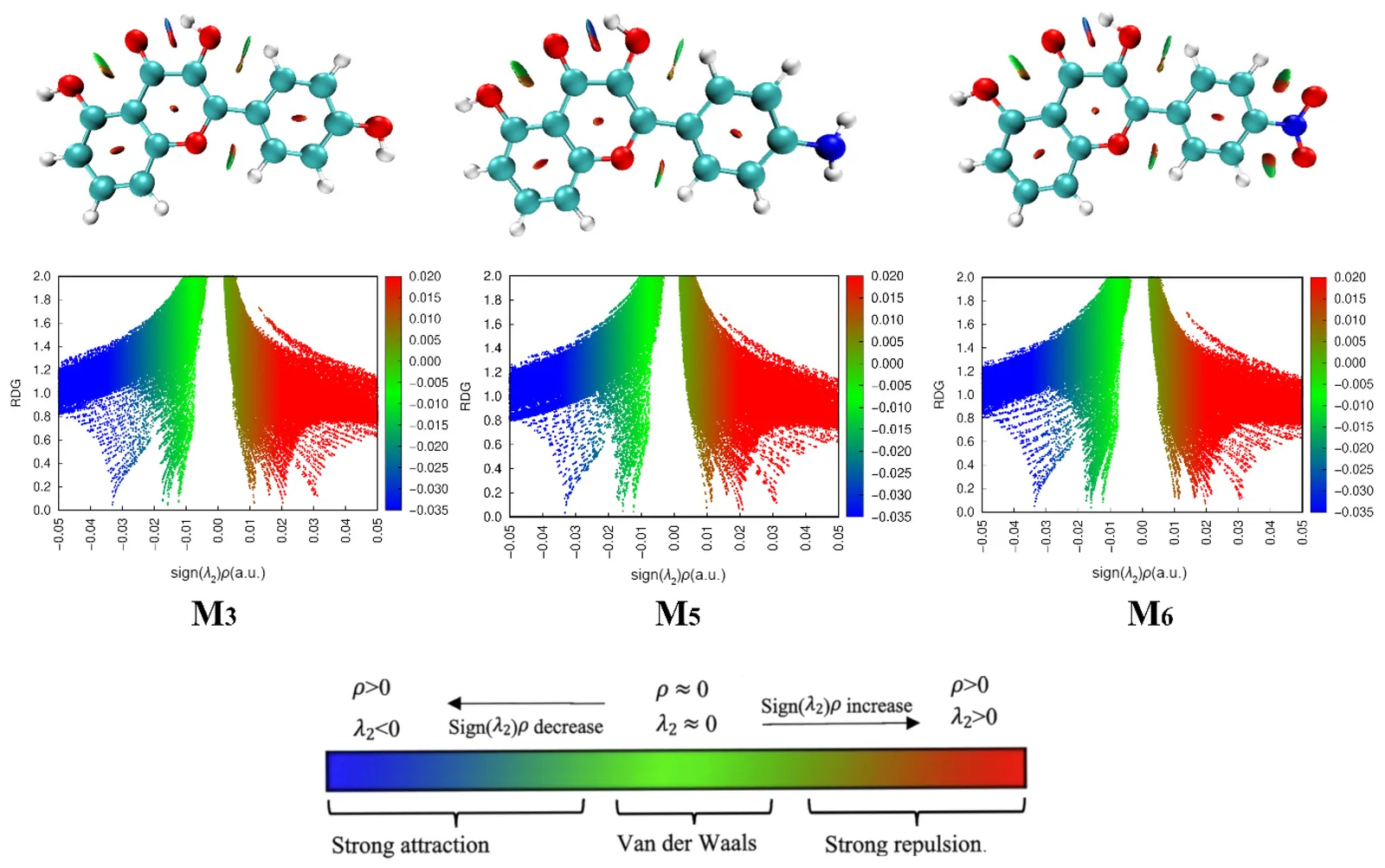
RDG-NCI (2D and 3D plots) of M_3_, M_5_ and M_6_.

The RDG is a powerful computational method used to visualize and quantify non-covalent interactions (NCIs) within molecular systems [68]. It helps distinguish between different types of interactions. These calculations were conducted using Multiwfn 3.8 software [69]. From Figure 12, the color-coded isosurface (2D plot) representation highlights different interaction regions: blue regions correspond to strong attractive interactions, such as hydrogen bonding, green regions indicate weak van der Waals interactions (vdW), and red regions suggest steric repulsion due to spatial hindrance. The presence of extended green zones implies significant dispersion interactions, contributing to the overall molecular stability. Meanwhile, the localized blue regions confirm the existence of hydrogen bonds, which play a crucial role in stabilizing the molecular conformation. Conversely, the red zones, primarily observed in sterically crowded areas, indicate repulsive interactions that may affect the molecular geometry and reactivity. The obtained results confirm the existence of a balance between attractive and repulsive forces, which plays a fundamental role in the reactivity and stability of the investigated molecules. In the 3D plot (Figure 12), denser regions signify strong interactions, whereas speckled regions suggest weaker interactions [70]. The distinct red-spotted zones are concentrated within aromatic rings, marking areas of intense steric repulsion. In contrast, a relatively thick blue zone appears between the hydrogen atom bonded to oxygen and the carbonyl oxygen atoms across all molecules, signifying strong attractive interactions. Meanwhile, the green regions observed in the three molecules highlight the presence of vdW forces.

### 3.5. Molecular Dynamics Simulations

**Figure 13:**
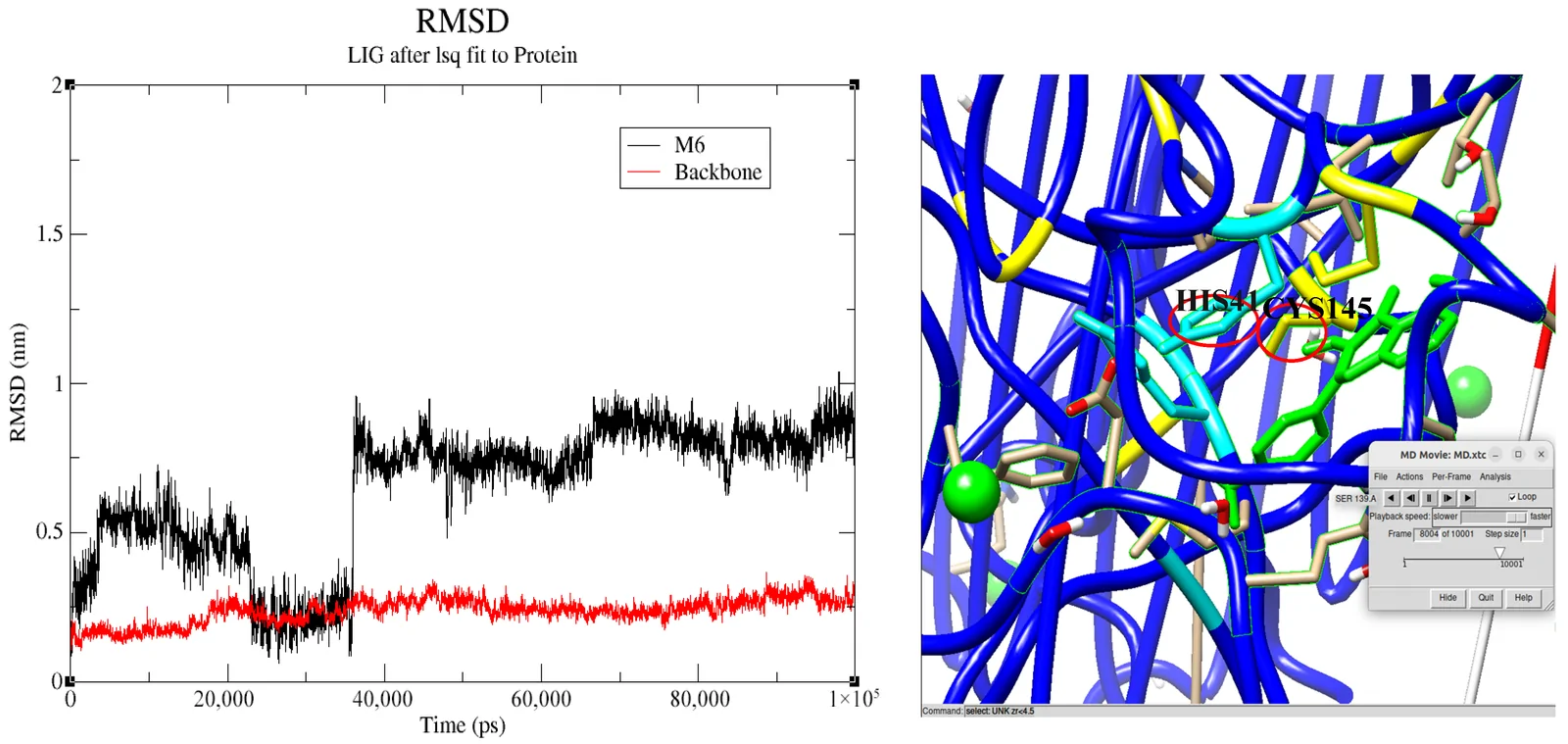
Protein-backbone and ligand RMSD profiles show a late M6 reorientation followed by a plateau while the ligand remains in the M^pro^ active-site region.

Docking prioritized M6 within the designed series and placed its predicted pose near the M^pro^ active-site residues His41 and Cys145; this model result motivated the molecular dynamics analysis. Building on these promising findings, a molecular dynamics (MD) simulation was carried out to thoroughly assess the stability of the ligand–protein interaction at the atomic level and to observe the temporal evolution of specific interactions. This approach is particularly valuable, as it not only evaluates the duration and robustness of the bonds identified during docking but also identifies any conformational alterations that may affect M_6_’s inhibitory efficacy. By incorporating dynamic parameters such as thermal fluctuations, side-chain mobility in the active site, and explicit solvation of the complex, the simulation provides a more accurate and realistic picture of how M6 behaves within the viral protease. Moreover, a detailed examination of the RMSD traces from the MD simulations (Figure 13) highlights multiple stages in the conformational pathway of M_6_ relative to the M^pro^ backbone. From 0 to approximately 22 ns, ligand RMSD rises as M6 explores orientations relative to its starting pose. Between approximately 22 and 35 ns, the ligand RMSD transiently approaches the protein-backbone trace. After approximately 35 ns, ligand RMSD increases to a plateau near 0.75 nm. This plateau indicates that M6 adopts a pose substantially displaced from the docked reference. Visual inspection of the 80 ns snapshot showed M6 retained within the active-site region, approximately 4.5 Å from His41/Cys145 [71,72]. Because no trajectory-clustering output was available, this transition is described as a late reorientation rather than proof of a unique adapted state; the interpretation is limited to the available distance and late-window energetic evidence.

**Figure 14:**
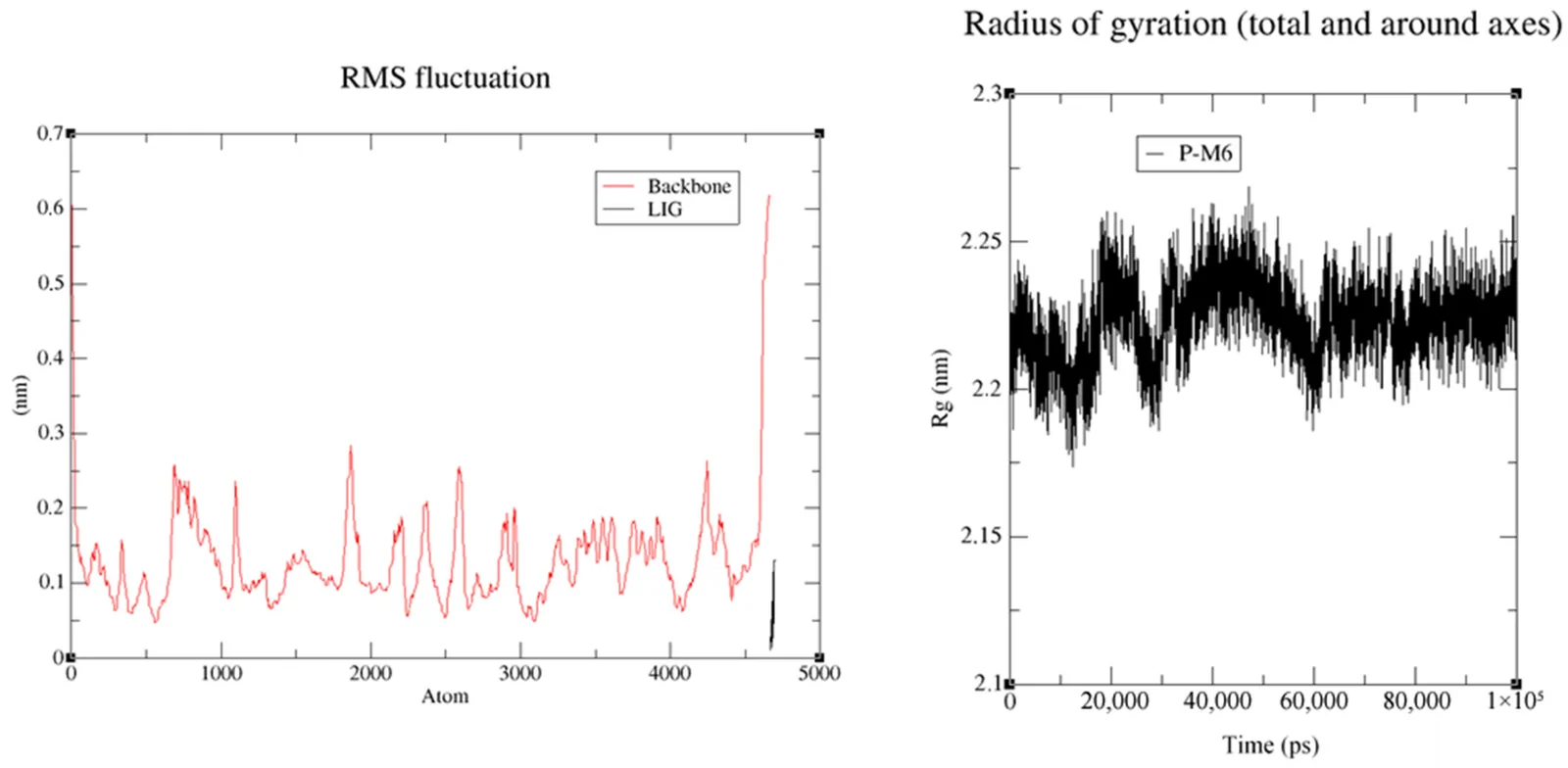
RMSF and radius-of-gyration (Rg) profiles describing local protein flexibility and preservation of global M^pro^ compactness during the M6 simulation.

The joint evaluation of RMSF and radius of gyration (Rg) displayed in Figure 14 provides a panoramic assessment of the SARS-CoV-2 protease-M_6_ ligand complex’s dynamic and structural integrity during the molecular simulation. RMSF measurements show moderate, localized backbone fluctuations in flexible segments, while Rg remains around 2.20–2.25 nm, indicating preservation of global protein compactness [73].

**Figure 15:**
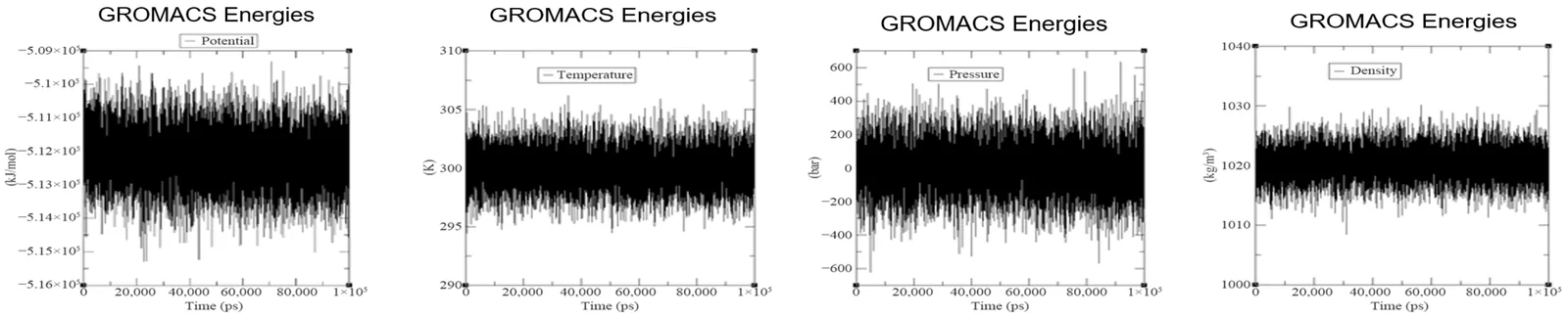
Potential energy, temperature, pressure, and density profiles used to assess thermodynamic control of the M^pro^-M6 molecular dynamics simulation.

The energy plots obtained from the molecular dynamics (MD) simulation (Figure 15) provide strong evidence of the system’s overall stability. Potential energy, temperature, pressure, and density fluctuate around stable mean values across the 100 ns trajectory, supporting adequate thermodynamic control of the simulated system [74]. Specifically, the potential energy shows only minor oscillations around a stable average value, signifying a robust energetic profile for the system. Likewise, the temperature remains near 300 K, confirming that the simulation conditions are suitably maintained and that no significant thermal fluctuations occur that might affect the complex’s molecular dynamics. Pressure fluctuations hover around 0 bar, indicating that the system is well-balanced under standard atmospheric conditions, while the density remains stable and close to the expected value for biological systems (~1020 kg/m^3^), confirming the physical soundness of the complex’s solvation environment.

### 3.6. MM/GBSA Free-Energy Analysis

MM/GBSA calculations were carried out for the M6-SARS-CoV-2 main protease (M^pro^) complex using the 9C8Q-derived receptor to estimate the binding free energy (ΔG_bind_, kcal·mol^−1^). A single-trajectory protocol was applied to 100 equilibrated snapshots uniformly harvested from the 75–100 ns window of the MD production (spacing 0.25 ns). This window was chosen to characterize the final ligand pose rather than average across the initial exploration and conformational transition. The total free energy was expressed as the sum of gas-phase molecular mechanics terms, van der Waals and Coulombic electrostatics (ΔG_gas_ = ΔE_vdW_ + ΔE_ele_), and solvent responses comprising the polar Generalized-Born and non-polar surface-area components (ΔG_solv_ = ΔG_GB_ + ΔG_surf_); conformational entropy was not included; the estimates are therefore interpreted comparatively and not as absolute experimental binding free energies [75].

$$\Delta G_{\mathrm{bind}\;}=\Delta G_{\mathrm{gas}\;}+\Delta G_{\mathrm{solv}\;}=\left(\Delta E_{\mathrm{vdW}\;}+\Delta E_{\mathrm{elec}\;}\right)+\left(\Delta G_{GB}+\Delta G_{\mathrm{surf}\;}\right)\left(kcal\cdot {mol}^{-1}\right)$$

**Figure 16:**
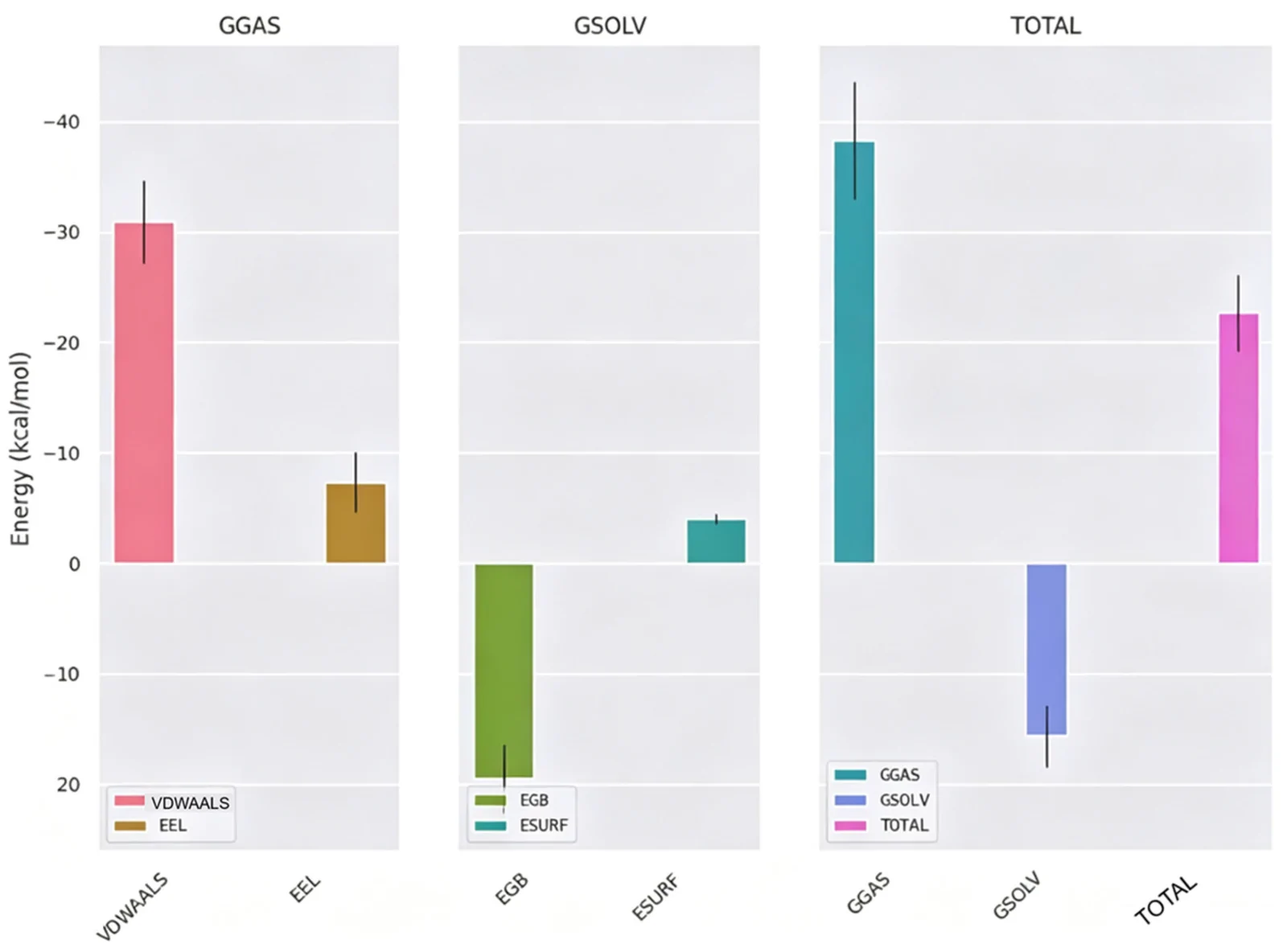
MM/GBSA energy decomposition of the M6-SARS-CoV-2 M^pro^ complex showing gas-phase (GGAS), solvation (GSOLV), and total binding free energy contributions.

Figure 16 reports a favorable average late-window MM/GBSA estimate for the M6-SARS-CoV-2 M^pro^ complex of −22.65 kcal·mol^−1^ (100 snapshots). Binding is dominated by van der Waals stabilization (ΔE_vdW_ = −30.91 kcal·mol^−1^), complemented by a modest electrostatic contribution (ΔE_elec_ = −7.31 kcal·mol^−1^). Opposing this, the polar solvation term is destabilizing (ΔG_GB_ = +19.58 kcal·mol^−1^), while nonpolar surface burial is favorable (ΔG_surf_ = −4.01 kcal·mol^−1^). Altogether, Figure 16 depicts a hydrophobically driven, well-packed pose in which dispersion contacts outweigh desolvation costs, supporting a favorable late-window MM/GBSA estimate within the stated model.

**Figure 17:**
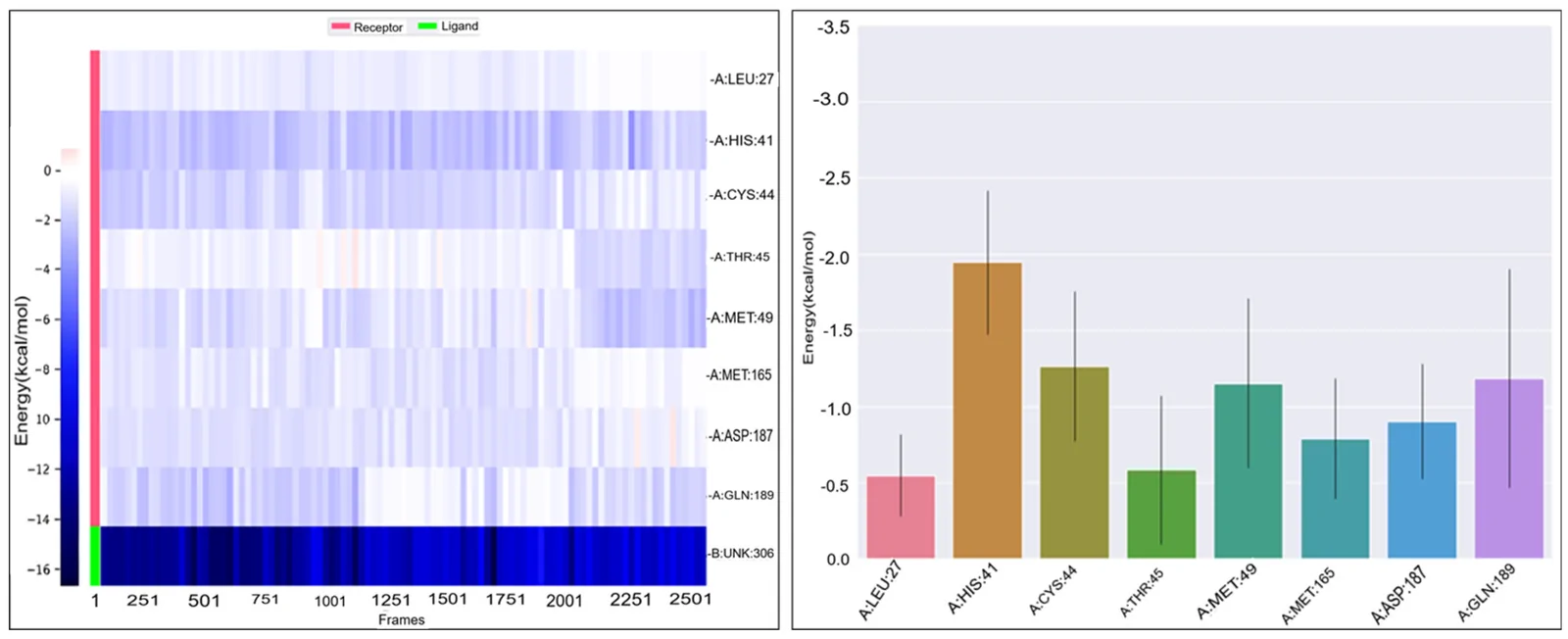
Time-resolved residue–ligand interaction-energy heatmap and mean per-residue energetic contributions for the M6-M^pro^ complex during the 75–100 ns analysis window. The heatmap shows the sampled late-trajectory frames; bars report mean values with variability from the 100 MM/GBSA snapshots.

To examine whether the late ligand RMSD plateau retained favorable active-site interactions, per-residue MM/GBSA decomposition was evaluated over 75–100 ns (Figure 17). The time-resolved heatmap shows predominantly negative residue–ligand interaction energies across the late trajectory. His41 provides the largest mean favorable contribution (approximately −1.9 kcal mol^−1^), followed by Cys44, Gln189, and Met49 (approximately −1.1 to −1.3 kcal mol^−1^); Met165 and Asp187 also contribute favorably, whereas Leu27 and Thr45 are weaker contributors. Thus, the MM/GBSA per-residue decomposition provides additional energetic support for the persistence of favorable interactions between M6 and key catalytic-pocket residues during the late stage of the simulation, indicating that the reoriented ligand pose maintained relatively stable energetic engagement with the active-site environment.

## 4. Conclusions

In summary, the in silico campaign shows that para-substitution differentiates the predicted drug-likeness, toxicity liabilities, docking behavior, and electronic properties of the flavonoid series. Among M1–M7, M6 is prioritized only as a computational lead because it was the best-ranked designed analogue in docking and retained favorable late-window energetic contributions after a substantial MD reorientation; its strong predicted mutagenicity remains a major liability. Complementary DFT calculations reveal M6 possesses the narrowest HOMO-LUMO gap and highest electrophilicity in the series. Collectively, the results justify biochemical M^pro^ inhibition assays, cellular antiviral testing, cytotoxicity evaluation, and focused genotoxicity studies before any efficacy or safety conclusion.

## Figures and Tables

**Table 1:** SwissADME-assessed physicochemical and pharmacokinetic profiles of the molecules.

Compound	M1	M2	M3	M4	M5	M6	M7	Lopinavir
Molecular weight (g mol^−1^)	254.24	268.26	270.24	284.26	269.25	299.24	279.25	628.8
H-bond acceptors	4	4	5	5	4	6	5	5
H-bond donors	2	2	3	2	3	2	2	4
Rotatable bonds	1	1	1	2	1	2	1	17
TPSA (Å^2^)	70.67	70.67	90.9	79.9	96.69	116.49	94.46	120
CLogP	2.6	2.73	1.99	2.4	1.85	1.67	2.07	4.51
Lipinski: violations	0	0	0	0	0	0	0	1
Veber: violations	0	0	0	0	0	0	0	1
Ghose: violations	0	0	0	0	0	0	0	3
Bioavailability score	0.55	0.55	0.55	0.55	0.55	0.55	0.55	0.55

**Table 2:** ADMETlab 3.0 model outputs for M1–M7 and lopinavir.

Compounds	M1	M2	M3	M4	M5	M6	M7	Lopinavir
HIA-positive probability (%)	0.79	1.3	2.5	20	1	2.1	37	0.0242
Caco-2 permeability [log10(Papp, cm/s)]	−4.86	−4.92	−5.30	−5.04	−5.38	−5.19	−5.03	−5.59
MDCK permeability [log10(Papp, cm/s)]	−4.729	−4.75	−4.81	−4.76	−4.89	−4.69	−4.73	−5.056
PAMPA high-permeability probability (0–1)	0.40	0.55	0.78	0.33	0.65	0.83	0.61	0.003
P-gp inhibitor probability (0–1)	0.96	0.97	0.39	0.86	0.17	0.79	0.97	0.999
P-gp substrate probability (0–1)	0.05	0.076	0.11	0.11	0.25	0.02	0.006	0.95
OATP1B1 inhibitor probability (0–1)	0.92	0.97	0.95	0.97	0.9	0.66	0.89	0.99
OATP1B3 inhibitor probability (0–1)	0.99	0.99	0.99	0.99	0.99	0.98	0.99	0.52
BCRP inhibitor probability (0–1)	0.91	0.63	0.88	0.90	0.32	0.79	0.97	0.31
BSEP inhibitor probability (0–1)	0.98	0.99	0.84	0.93	0.15	0.84	0.99	0.999
MRP1 inhibitor probability (0–1)	0.91	0.94	0.87	0.82	0.65	0.99	0.03	0.0009
BBB-penetration probability (0–1)	0.076	0.05	0.003	0.005	0.014	0.0015	0.002	2.56 × 10^−5^
PPB (%)	98.94	98.85	98.52	98.54	98.21	98.30	98.11	97.94
logVDss [log10(L/kg)]	−0.74	−0.73	−0.89	−0.70	−0.82	−0.59	−0.34	−0.44

**Table 3:** Predicted CYP450 inhibitor and substrate probabilities for M1–M7 and lopinavir.

Compounds	M1	M2	M3	M4	M5	M6	M7	Lopinavir
CYP1A2-inh	0.98	0.95	0.97	0.44	0.38	0.79	0.41	1.57 × 10^−7^
CYP1A2-sub	0.28	0.14	0.22	0.47	0.031	0.93	0.35	0.005
CYP2C19-inh	0.87	0.51	0.31	0.084	0.052	0.156	0.364	0.78
CYP2C19-sub	0.001	0.003	0.003	0.002	0.002	0.004	0.0001	0.91
CYP2C9-inh	0.98	0.99	0.97	0.98	0.8	0.91	0.98	0.036
CYP2C9-sub	0.99	0.99	0.98	0.99	0.98	0.98	0.96	0.0002
CYP2D6-inh	0.017	0.00005	0.0009	0.0008	0.00002	0.00006	0.00002	0.0007
CYP2D6-sub	0.91	0.9	0.93	0.99	0.65	0.7	0.64	0.012
CYP3A4-inh	0.0006	0.005	0.05	0.002	0.08	0.01	7.21 × 10^−5^	0.98
CYP3A4-sub	0.01	0.72	0.01	0.43	0.046	0.11	0.34	0.97

**Table 4:** Docking scores and residue-specific hydrogen-bond distances for M1–M7, lopinavir, and the 9C8Q co-crystallized ligand.

Compounds	Docking Score (kcal mol^−1^)	Hydrogen-Bond Residue (s)	Residue-Distance Pair (s) (Å)
**M_1_**	−7.2	His163, Met165	His163: 3.28; Met165: 1.78
**M_2_**	−7.5	His163, His164, Met165	His163: 1.83; His164: 2.52; Met165: 3.27
**M_3_**	−7.6	Phe140, His163, His164	Phe140: 1.98; His163: 1.79, 2.85; His164: 2.21
**M_4_**	−7.7	Leu141, Cys145, His163, Glu166	Leu141: 2.13; Cys145: 2.60; His163: 2.28, 2.76; Glu166: 2.97
**M_5_**	−7.6	Leu141	Leu141: 1.86
**M_6_**	−8.1	His41, Cys44, Phe140, His163, Met165	His41: 2.64; Cys44: 3.53; Phe140: 1.96; His163: 1.79; Met165: 3.34
**M_7_**	−7.2	Cys44, His163, His164, Phe140	Cys44: 3.65; His163: 1.88; His164: 1.89; Phe140: 2.08
**Lopinavir**	−9.2	Glu166, Thr190	3.71 (one distance value reported for Glu166/Thr190)
**Co-crystal**	−8	His163	His163: 1.91

**Table 5:** Calculated HOMO, LUMO and band gap energies for M_3_, M_5_ and M_6_.

Compounds	$E_{HOMO}$	$E_{LUMO}$	$E_{gap}$
M_3_	−5.636	−1.870	3.766
M_5_	−5.320	−1.714	3.606
M_6_	−6.345	−2.903	3.442

**Table 6:** The estimated values of reactivity parameters of M_3_, M_5_ and M_6_.

Compounds	$E_{HOMO}$ (eV)	$E_{LUMO}$ (eV)	μ (eV)	χ (eV)	η (eV)	S (${eV}^{-1}$)	ω (eV)
M_3_	−5.636	−1.870	−3.753	3.753	1.883	0.265	3.74
M_5_	−5.320	−1.714	−3.517	3.517	1.803	0.277	3.43
M_6_	−6.345	−2.903	−4.624	4.624	1.721	0.290	6.21

## Data Availability

All data generated or analysed during this study are available within the article and in the public GitHub repository: https://github.com/khibech/para-Substituted-Flavonoids- (accessed on 24 August 2026). The repository contains the research data supporting the transparency and reproducibility of the study and was created on 14 June 2025.

## Abbreviations

HIA %	Human Intestinal Absorption
Caco-2	Caco-2 cell-monolayer permeability
MDCK	Madin–Darby canine kidney cells
PAMPA	Parallel Artificial Membrane Permeability Assay
P-gp_inh	P-glycoprotein Inhibitor Probability
P-gp_sub	P-glycoprotein Substrate Probability
OATP1B1	Organic Anion Transporting Polypeptide 1B1
OATP1B3	Organic Anion Transporting Polypeptide 1B3
BCRP	Breast Cancer Resistance Protein
BSEP	Bile Salt Export Pump
MRP1	Multidrug Resistance-associated Protein 1
BBB	Blood–Brain Barrier permeability
PPB %	Plasma Protein Binding Percentage
